# Unveiling antiplasmodial alkaloids from a cumulative collection of *Strychnos* extracts by multi-informative molecular networks

**DOI:** 10.3389/fmolb.2022.967012

**Published:** 2022-09-26

**Authors:** Olivier Bonnet, Mehdi A. Beniddir, Pierre Champy, Gilles Degotte, Lúcia Mamede, Pauline Desdemoustier, Allison Ledoux, Alembert Tiabou Tchinda, Luc Angenot, Michel Frédérich

**Affiliations:** ^1^ Laboratory of Pharmacognosy, Center of Interdisciplinary Research on Medicines (CIRM), University of Liege, Liege, Belgium; ^2^ Équipe “Chimie des Substances Naturelles” BioCIS, CNRS, Université Paris-Saclay, Châtenay-Malabry, France; ^3^ Institute of Medical Research and Medicinal Plants Studies (IMPM), Yaoundé, Cameroon

**Keywords:** Loganiaceae, *Strychnos*, malaria, antiplasmodial, mass spectrometry, metabolomics, chemoinformatics, molecular networking

## Abstract

Malaria, a disease known for thousands of years and caused by parasites of the *Plasmodium* genus, continues to cause many deaths throughout the world today, particularly due to the emergence of parasite resistance to the current therapeutic arsenal. Plants of the *Strychnos* genus, remarkable due to their multiple traditional uses as well as their alkaloid content, are promising candidates to develop new antimalarial treatments. Indeed, previous research on this plant group has shown promising (≤ 5 µg/ml) or good (between 5 and 15 µg/ml) antiplasmodial activities. Using the chloroquine-sensitive strain of *Plasmodium falciparum* (3D7), and artemisinin as positive control, a screening of antiplasmodial activities from 43 crude methanolic extracts from 28 species of the *Strychnos* genus was carried out in three independent assays. A total of 12 extracts had good (6 extracts) or promising (6 extracts) antiplasmodial activities. These results allowed both to confirm known activities but also to detect new ones. These extracts were then analyzed by HPLC-ESI(+)-Q/TOF, and the processed MS/MS data allowed to generate a molecular network in which the antiplasmodial activities were implemented as metadata. The exploration of the molecular network revealed the presence of alkaloids still unknown, and potentially active against malaria, in particular alkaloids close to usambarensine and its derivatives. This study shows that the emergence of molecular networking offers new leads for identifications of alkaloids from the *Strychnos* genus. The presence of unknown alkaloids potentially active against malaria confirms all the interest to continue in studying the *Strychnos* genus. Bioassay- and mass-guided fractionations as well as various dereplication tools would allow to identify and characterize these interesting alkaloids further.

## Introduction

The pantropical family of Loganiaceae is divided into four tribes: Antonieae Endl., Loganieae Endl., Spigelieae Dumort, and Strychneae Dumort. A total of 16 genera, including 460 species, are distributed in these different tribes. Nearly half of these species (approximately 200) are in the *Strychnos* genus, that belongs to the tribe Strychneae Dumort. The plants of the *Strychnos* genus are distributed in different continents, namely Africa (75 species), Asia and Oceania (about 44 species), and Central and South America (at least 73 species) (Bisset, 1970; Krukoff, 1972; Bisset, 1974; Setubal et al., 2021; World Flora Online, 2022).

Regarding the pharmacology of the *Strychnos* genus, emphasis in the past was on the investigation of the alkaloids present in tetanizing or curarizing species. Indeed, many *Strychnos* species are the classic base poisons of South American as well as South-East Asian arrow and blowpipe poisons (Krukoff, 1972; Bisset, 1974). The place of *Strychnos* in the hierarchy of African hunting poisons is secondary and their use limited very locally. So, the Banyambo, a small tribe located in Tanzania, produce a complex curarizing poison of which the roots of *Strychnos usambarensis* play an important part (Angenot, 1971). On the contrary, the importance of *Strychnos icaja* as a trial by ordeal poison is higher and known a long time ago in several countries of West and central Africa (Gabon, Congo, Democratic Republic of the Congo, Central African Republic) (Philippe et al., 2004).

However, it is evident that the plants of many African *Strychnos* species are also well known for their multiple traditional uses (Bisset, 1970). Indeed, they are used to treat snakebites, arthritis, rheumatism, asthma, bronchitis, diarrhea, hemorrhoids, dyspepsia, fever, and many others (Dr. Duke’s Phytochemical and Ethnobotanical Databases, 2021). All these traditional uses make these plants interesting candidates in the development of new drug treatments. That is why the *Strychnos* genus was the subject of numerous research works in our laboratory for more than three decades, especially for its promising antiplasmodial (*in vitro*) and antimalarial (*in vivo*) properties. During these investigations, many isolated monoterpene indole alkaloids showed promising activities on various chloroquine-sensitive (CQS) strains as well as on some chloroquine-resistant (CQR) strains (Wright et al., 1994; Wright et al., 1996; Frédérich et al., 1999; Frédérich et al., 2000; Frédérich et al., 2001; Frédérich et al., 2002; Frédérich et al., 2003; Tchinda et al., 2012; Tchinda et al., 2014). Among the most active compounds on CQS strains, we could cite strychnogucine B (0.6170 µM ± 0.067) and strychnohexamine (1.097 µM ± 0.099) both alkaloids isolated from *S. icaja* roots. Against CGR strains, we could give as examples 3′,4′-dihydrousambarensine (0.032 µM ± 0.002) and isostrychnopentamine, both isolated respectively from roots and leaves of *S. usambarensis* without forgetting strychnogucine B (0.085 µM ± 0.01) and 18-hydroxyisosungucine (0.14 µM ± 0.046) from *S. icaja* roots (Frédérich et al., 1999; Frédérich et al., 2001; Philippe et al., 2002). Other papers described *in vitro* and *in vivo* screenings with crude extracts of various *Strychnos* species (e.g., Philippe et al., 2005; Philippe et al., 2007; Fentahun et al., 2017).

Malaria (from the Italian *mal’aria* meaning “bad air”) is a widespread disease in the world that is known for thousands of years. In fact, Indian texts dating back to the sixth century ACN described the symptoms of malaria infection. This disease, transmitted by the bites of female mosquitoes of the *Anopheles* genus, is caused by a protozoan parasite of the Apicomplexa phylum, and more precisely from the *Plasmodium* genus. Five species are capable of infecting humans: *Plasmodium falciparum*, *Plasmodium vivax*, *Plasmodium ovale*, *Plasmodium malariae*, and *Plasmodium knowlesi* (Cox, 2010). To fight this disease, different hygienic and dietary advices are applied such as wearing long clothes or using insecticide impregnated mosquito nets. In addition, a variety of antimalarial treatments were developed such as artemisinin-based treatments. These treatments led to a reduction in the number of cases. However, the parasites began to show increasing resistance to these drugs, preventing the goal of total eradication of this disease. Malaria remains a major public health problem today, especially in Africa where it is devastating. Indeed, in Africa, between 2019 and 2020, malaria cases increased from 213 million to 228 million, and deaths caused by malaria increased from 534,000 to 602,000 (World Health Organization, 2021a; World Health Organization, 2021b; World Health Organization, 2022).

In the face of this uncontrollable situation, researchers are conducting important research to find new molecules to develop new antimalarial treatments. Expanding the therapeutic arsenal would allow to counter the growing resistance of the parasites and potentially eradicate the pathology. In view of their promising antiplasmodial and antimalarial potential, plants of the *Strychnos* genus are therefore very interesting candidates.

In recent years, the field of natural products research has evolved significantly with the development of various dereplication tools, including molecular networking, which allows the quick discrimination of known and unknown metabolites based on comparisons with databases of mass spectra (Yang et al., 2013; Wang et al., 2016; Nothias et al., 2018; Fox Ramos et al., 2019a; Fox Ramos et al., 2019b; Beniddir et al., 2021). Inspired by these advances, we envisioned that the use of molecular networks would be an efficient approach to both explore the chemodiversity of alkaloids in plants of the *Strychnos* genus but also to target metabolites that have not yet been identified in the previous studies.

In this context, the objectives of this study were first to screen the antiplasmodial activities of methanolic crude extracts from a large number of *Strychnos* species, to explore then by molecular networking the chemodiversity of monoterpene indole alkaloids, and to finally apply as metadata in the molecular network the antiplasmodial activities obtained with the aim of detecting and targeting molecular families containing unknown alkaloids, potentially active against malaria.

## Materials and methods

### Materials, chemicals, and reagents

The twenty-eight species of *Strychnos*, preserved in the collections of the University of Liege (Belgium), were collected in several countries: Rwanda, Congo, Zimbabwe, Tanzania, Cameroon, India, Cambodia, and Brazil. These species were *S. usambarensis* Gilg ex Engl., *S. variabilis* De Wild., *S. gossweileri* Exell, *S. mellodora* S.Moore, *S. phaeotricha* Gilg, *S. brasiliensis* (Spreng.) Mart., *S. innocua* Delile, *S. henningsii* Gilg, *S. angolensis* Gilg, *S. scheffleri* Gilg, *S. tricalysioides* Hutch. & M.B.Moss, *S. spinosa* Lam., *S. longicaudata* Gilg, *S. malchairi* De Wild., *S. mattogrossensis* S.Moore, *S. icaja* Baill., *S. nux-vomica* L., *S. ignatii* P.J. Bergius, *S. potatorum* L.f., *S. malacoclados* C.H. Wright, *S. camptoneura* Gilg and Busse, *S. congolana* Gilg, *S. boonei* De Wild., *S. staudtii* Gilg, *S. elaeocarpa* Gilg ex Leeuwenberg, *S. densiflora* Baill., *S. tchibangensis* Pellegr., and *S. johnsonii* Hutch. & M.B.Moss. All information about the samples used in this study is presented in the Supplementary Table S1. Each sample was dried at 40°C, stored dry at moderate room temperature, and protected from light. Some samples were collected several years ago. The question of the stability of alkaloids was therefore raised. Numerous previous studies of alkaloids showed a high stability (Phillipson, 1982; Frédérich et al., 1998; Eloff, 1999; Soto-Sobenis et al., 2001; Harborne, 2012; Yilmaz, Nyberg, and Jaroszewski, 2012). Oxidation reactions cannot be excluded, but no examples were highlighted in the different studies.

Methanol and DMSO were purchased from VWR Chemicals BDH (Leuven, Belgium). Methanol of HPLC grade was obtained from Merck (Darmstadt, Germany). The solvents of UHPLC-MS grade (methanol and formic acid) came from Sigma-Aldrich (Overijse, Belgium). About milli-Q water, two systems were used: a milli-Q reference A+ system^®^ at the University of Liège and a MILLIPORE Synergy UV^®^ at Université Paris-Saclay. These two systems were purchased from Merck (Darmstadt, Germany).

### Sample preparation

A total of 44 samples, described in the Supplementary Table S1, were ground using an IKA A10 mill (Staufen, Germany) to obtain 10 g powder. Extractions were then performed in methanol using the SpeedExtractor E-914^®^ (Büchi, Hendrik-Ido-Ambacht, Netherlands). This device allowed extractions with pressurized solvents, which offers a better extraction. Moreover, by means of four cells each containing plant powder and sand, four samples were extracted at the same time during three extraction cycles. These cycles were composed of 1 min of heat-up time, 15 min of hold time, and 2 min of discharge time. Then, the system was washed with solvent for 2 min, and was dried with nitrogen for 3 min. The crude extracts collected were evaporated using Rotavapor^®^ and Multivapor^®^ (Büchi). In order to dry the extracts, they are placed in a vacuum oven (Heraeus, Hanau, Germany) for one night at room temperature.

### Antiplasmodial assays

*In vitro* cultures of *P. falciparum* in the asexual erythrocyte stage were maintained following the procedure of Trager and Jensen (1976). The strain of parasites 3D7 is a chloroquine-sensitive strain that was obtained from the Malaria Research and Reference Reagent Resource Center, MR4. The culture medium was composed of RPMI 1640 (Gibco, Fisher Scientific, Merelbeke, Belgium) containing NaHCO_3_ (32 mM), HEPES (25 mM), and L-glutamine. The host cells were human red blood cells (A+ or O+). The medium was supplemented with 1.76 g/L of glucose (Sigma-Aldrich, Overijse, Belgium), 44 mg/ml of hypoxanthin (Sigma-Aldrich, Overijse, Belgium), 100 mg/L of gentamicin (Gibco, Fisher Scientific, Merelbeke, Belgium), and 10% human pooled serum (A+ or O+), as previously described. Each crude extract was dissolved in DMSO at a concentration of 10 mg/ml. The solutions of crude extracts were then diluted in the culture medium: for each solution to test, two-fold dilutions were performed eight times on a 96-well plate. With this method, the highest concentration tested is 100 µg/ml. Moreover, each sample was tested in duplicate. As positive control for all assays, artemisinin (Sigma-Aldrich, Machelen, Belgium) was used at an initial concentration of 100 ng/ml. After leaving the parasites in incubation with the diluted solutions of the crude extracts for 48 h, the impact on parasite growth was revealed using SYBR Green, a DNA intercalating compound. The procedure was adapted from the method described in the article of Dery et al. (2015). The SYBR Green solution was diluted in a lysis buffer composed of TRIS buffer (Sigma-Aldrich, Overijse, Belgium), EDTA (Merck, Darmstadt, Germany), saponin (Alfa Aesar, Karlsruhe, Allemagne), and triton (Merck, Darmstadt, Germany). Thus, 500 ml of lysis buffer contains 1.20 g TRIS buffer, 0.73 g EDTA, 40 mg saponin, and 0.4 ml triton. In order to reveal a plate, 2 µl of SYBR Green solution is diluted in 10 ml of lysis buffer. In new 96-well plates, 100 µl of solutions from assays are placed and 75 µL of SYBR Green is added. After 2 h of incubation, the plates are read with the FlexStation^®^ (Molecular Devices, Winnersh, United Kingdom) at 490 nm excitation wavelength and 530 nm emission wavelength. The half maximal inhibitory concentration (IC_50_) values were calculated from graphs. Averages of three IC_50_ values from three independent experiments (*n* = 3) performed on different days were calculated.

### Mass spectrometry analyses

A total of forty-three methanolic crude extracts were dissolved and ultrasonicated in methanol of HPLC grade (high-performance liquid chromatography). The concentration obtained was at 1 mg/ml. After transferring all the solutions into HPLC vials, they were injected into the HPLC-MS/MS system. The Agilent HPLC-MS system (Agilent Technologies, Massy, France) was composed of two modules: an Agilent 1,260 Infinity HPLC coupled to an Agilent 6,530 ESI-Q/TOF-MS (ElectroSpray Ionization Quadrupole Time of Flight Mass Spectrometry) operating in positive mode. The models were: G1367E for HiP sample, G1311B for quaternary pump, G1316A for column compartment, G6530A for TOF/QTOF mass spectrometer, and G4212B for DAD. The analytical column used was a SunFire^®^ C_18_ purchased from Waters (150 mm × 2.1 mm, 3.5 µm). The flow rate was at 250 µl/min. About the gradient, it was linear and variated from 5% B to 100% B in 30 min (A = Water + 0.1% formic acid; B = Methanol). The injection volume was 5 µl. The DAD detector was set at 210, 254, and 280 nm. For ESI conditions, the capillary temperature, the source voltage, and the sheath gas flow rate were set at 320°C, 3.5 kV, and 10 L/min, respectively. The mass spectrometer worked with the Extended Dynamic Range mode (2 GHz). Thanks to the divert valve, the first 3 min were eliminated. For every scan, 1 MS scan in positive mode was performed between *m/z* 100 and 1,200, and the five most intense ions were fragmented. Three fixed collision energies were applied at 30, 50, and 70 eV. The default charge was 1. The isolation width and minimum intensity were set at *m/z* 1.3 and 3,000 counts, respectively. Purine C_5_H_4_N_4_ [M + H]^+^ ion (*m/z* 121.0509) and hexakis (*1H*, *1H*, *3H*-tetrafluoropropoxy)-phosphazene C_18_H_18_F_24_N_3_O_6_P_3_ [M + H]^+^ ion (*m/z* 922.0098) were constituted the internal lock masses. The *m/z* values of the two internal calibrants were implemented in a permanent MS/MS list exclusion criterion in order to avoid that the signals from internal lock masses oversample the signals from samples analyzed.

### Feature-based molecular networking

The technique of feature-based molecular networking is described in the article of Nothias et al. (2020). First, the data obtained from the mass spectrometry analyses are converted into an “.mzXML” file (eXtensible Markup Language). The conversion was performed using MS Convert software edited by ProteoWizard (Chambers et al., 2012). The filter and the algorithm used were peak picking and vendor, respectively. The MS levels 1 and 2 were selected. Then, the “.mzXML” files were processed using MZmine 2 software (version 2.53) (Pluskal et al., 2010). Different stages of processing were carried out: suppression of noise (Method: Mass detection), creation of peak lists (Method: ADAP Chromatogram builder), deconvolution (Method: Chromatogram deconvolution), grouping of isotopes (Method: Isotopic peaks grouper), alignment (Method: Join aligner), gap-filling (Method: Same RT and *m/z* range gap filler), filtering (Method: Feature list rows filter), and export (Methods: Export to CSV file and Export/Submit to GNPS/FBMN). The intensities applied for the suppression of noises were the 5.8E3 and 5.0E1 for MS^1^ and MS^2^, respectively. For the creation of peak lists, a minimum of four points were necessary to build a peak. The intensity threshold was set at 5.8E3, and the *m/z* tolerance was *m/z* 0.02 and 10.0 ppm. About the deconvolution, the different settings applied were the following: the algorithm was wavelets (ADAP) (Myers et al., 2017) the *m/z* center calculation was auto, the retention time and *m/z* ranges for MS^2^ scan pairing were 1 min and 0.03 Da, respectively, the S/N threshold was 1, the S/N estimator was intensity window SN, the minimal feature height was 3,000, the coefficient/area threshold was 2, the peak duration range was 0.02–1.5 min, and the retention time wavelet range was 0.02 and 0.2 min. About the suppression of isotopes, 0.005, 15.0 ppm, and 0.5 min were the values for *m/z* retention time tolerances. The maximum charge was set at 1. The most intense peak corresponded to the representative isotope. All the peak lists were gathered according to the following criteria: the *m/z* and retention time tolerances were *m/z* 0.02, 15.0 ppm and 0.8 min, respectively, and the weights for *m/z* and retention time were 100 for the two settings. During the filtration step, only the MS/MS data were kept because they are essential to generate the molecular network. Moreover, during this step, the retention windows between 0–2.50 min and 45.59–49.83 min were deleted. Finally, the processed files are exported into “.mgf” without merging MS/MS spectra and “.CSV” formats to be imported into the GNPS platform (Global Natural Products Social Molecular Networking).

On the GNPS platform, the tolerances for precursor and fragment ions were set at 0.02 Da. The minimal cosine score for binding two metabolites was 0.65. For matching with the library, the threshold for the cosine score was 0.7. No filtration was applied, and the analogues were not searched. To highlight the active nodes against malaria, as metadata, a “.txt” file listing the antiplasmodial activities of the forty-three methanolic crude extracts was also imported.

### MolNetEnhancer annotation

The MS/MS data of the global molecular network were further annotated using MolNetEnhancer workflow exploiting exclusively the GNPS experimental annotations. MolNetEnhancer is a workflow that allow to have a more comprehensive chemical overview of metabolomics and to highlight structural details for each MS/MS spectra. Using the automated chemical classification through ClassyFire, the classifications provided by MolNetEnhancer were of different levels: from kingdoms to subclasses (Ernst et al., 2019).

## Results and discussion

### Methanolic extractions and yields

Forty-three methanolic crude extracts were obtained from different parts of twenty-eight *Strychnos* species. All the weightings and yields are presented in Supplementary Table S2. The yields were between 0.55% w/w and 25.92% w/w.

### Antiplasmodial assays of methanolic crude extracts from *Strychnos* spp.

**FIGURE 1:**
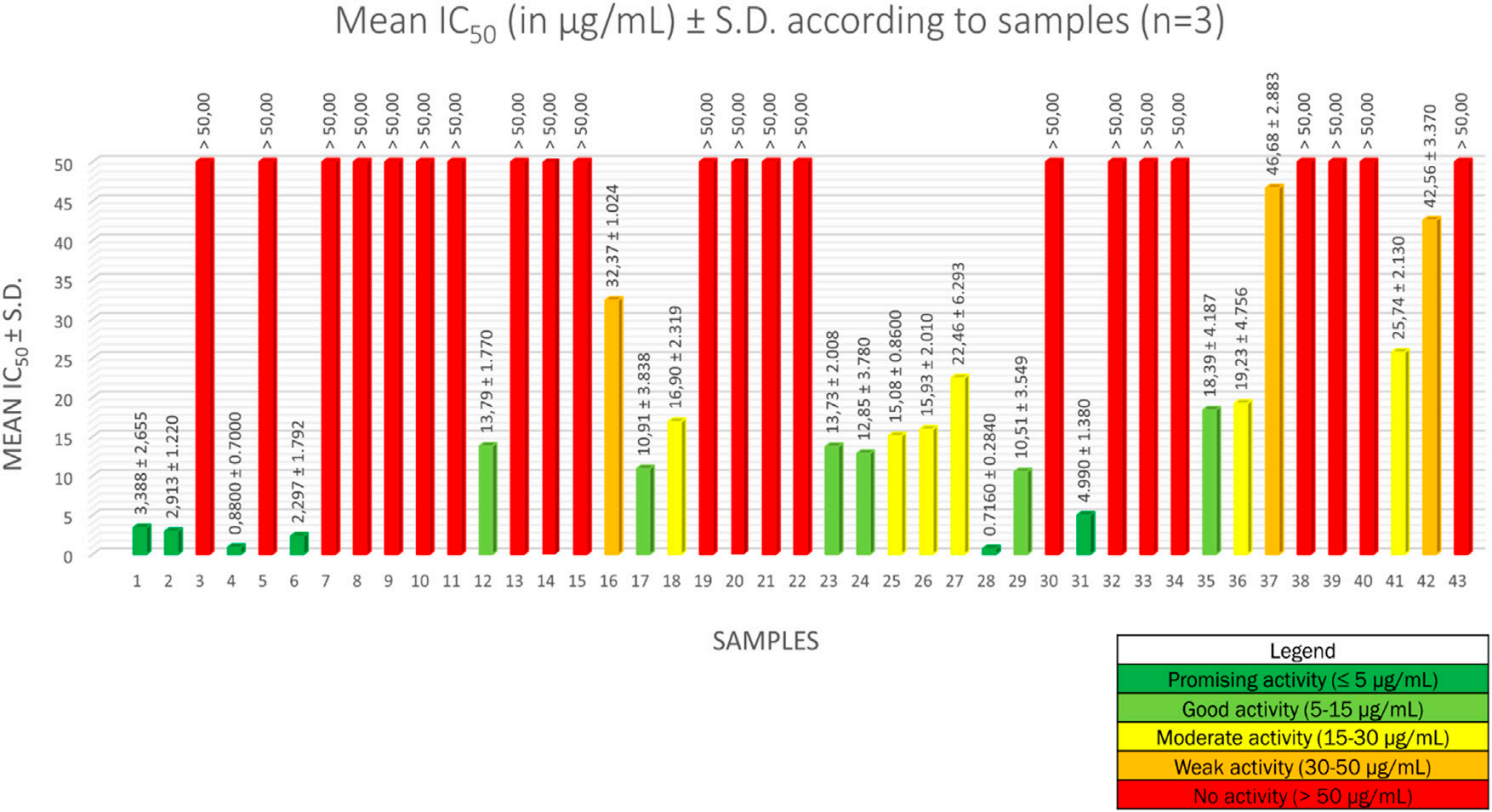
Mean IC_50_ values of 43 methanolic crude extracts of 28 *Strychnos* species.

The half maximal inhibitory concentration (IC_50_) values for the forty-three methanolic crude extracts are described in Figure 1. Sample number identifications are described in Supplementary Table S1. Results are averages expressed in µg/ml ± standard deviation (S.D.) of IC_50_ values of three independent experiments. For artemisinin, the mean IC_50_ is 3.092 ± 1.570 ng/ml, which means that the IC_50_ values obtained are valid (Ledoux et al., 2017).

To interpret the antiplasmodial activity levels, we implemented the following classification, based on the World Health Organization guidelines (Jansen et al., 2012): an activity ≤ 5 µg/ml was considered as a promising antiplasmodial activity, between 5 µg/ml and 15 µg/ml as a good activity, between 15 µg/ml and 30 µg/ml as a moderate activity, between 30 µg/ml and 50 µg/ml as a weak activity, and ≥ 50 µg/ml as a lack of activity.

Among the forty-three methanolic crude extracts tested, 14% (6 out of 43) showed a promising antiplasmodial activity, 14% (6 out of 43) a good activity, 14% (6 out of 43) an intermediate activity, 7% (3 out of 43) a weak activity, and 51% (22 out of 43) a lack of activity. *Strychnos* species with promising and good antiplasmodial activities are the following: *S. usambarensis* Gilg ex Engl. leaves (November 2007 and August 2008), *S. usambarensis* Gilg ex Engl. root barks, *S. variabilis* De Wild. root barks, *S. phaeotricha* Gilg leaves, *S. angolensis* Gilg root barks, *S. longicaudata* Gilg trunk barks, *S. malchairi* De Wild. leaves, *S. icaja* Baill. roots, *S. icaja* Baill. collar barks, *S. nux-vomica* L. root barks, and *S. malacoclados* C.H. Wright root barks. While antiplasmodial activities from trunk barks of *S. longicaudata*, and from leaves of *S. phaeotricha* and *S. malchairi* were never reported in the literature, the other results confirmed the reported activities in previous studies (Frédérich et al., 1999; Philippe et al., 2005).

### Feature-based molecular networking of *Strychnos* spp. methanolic crude extracts and exploration of their alkaloid content

**FIGURE 2:**
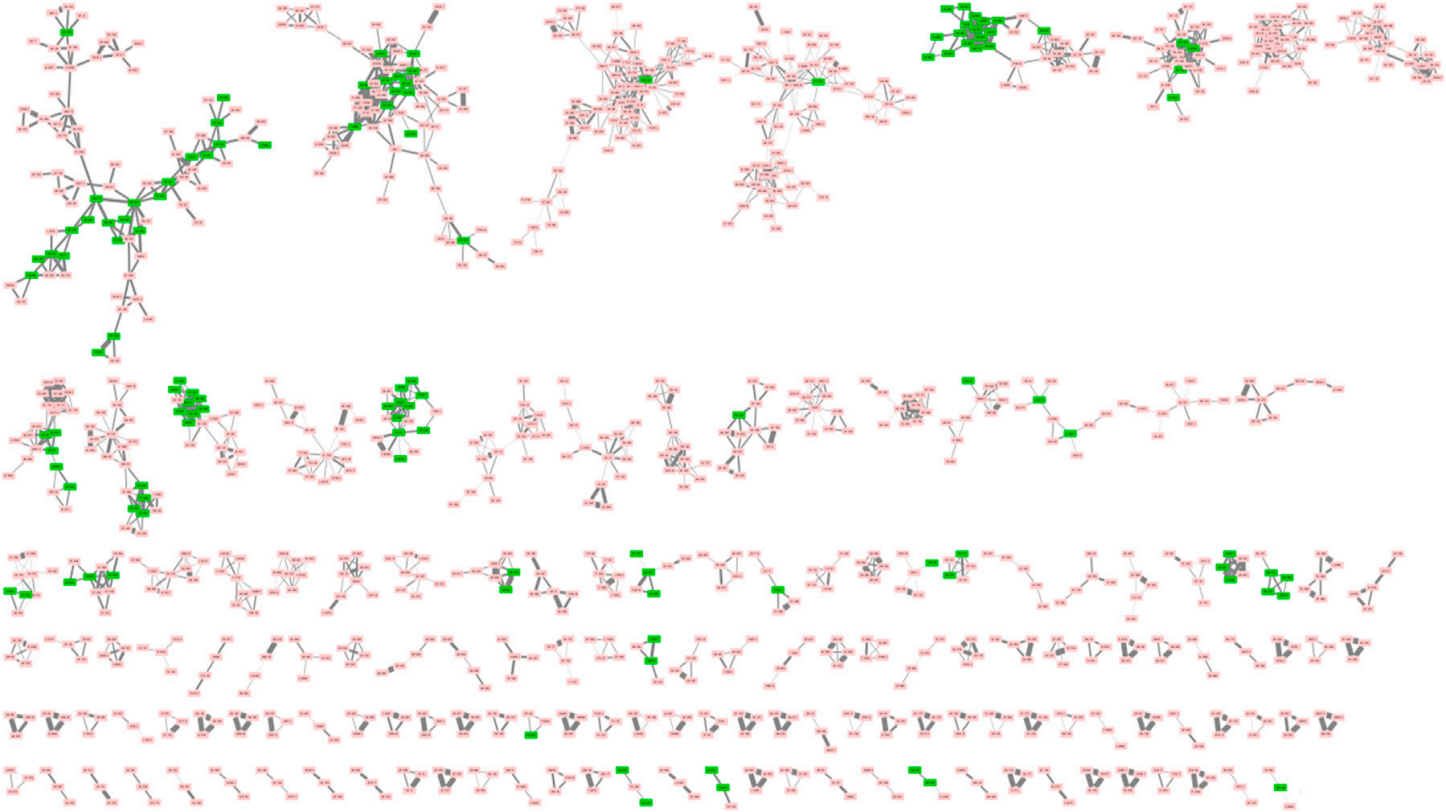
Part of molecular network of 28 *Strychnos* species. (Clusters with less than 3 nodes are not shown). The green nodes correspond to the annotated metabolites while the red nodes are the unannotated ones.

All processed data have been deposited on the GNPS platform by using the Feature-Based Molecular Networking (FBMN) workflow. The molecular network obtained contained 5,904 nodes, including 105 unique annotations (Figure 2). The following link provides access to the job and the molecular network: https://gnps.ucsd.edu/ProteoSAFe/status.jsp?task=880ec1b92b6d4cd1b73a225f2ab3dcdb. Different monoterpene indole alkaloids, such as 3′,4′-dihydrousambarensine, strychnine, icajine, strychnofoline, sungucine, were annotated thanks to the GNPS libraries.

**FIGURE 3:**
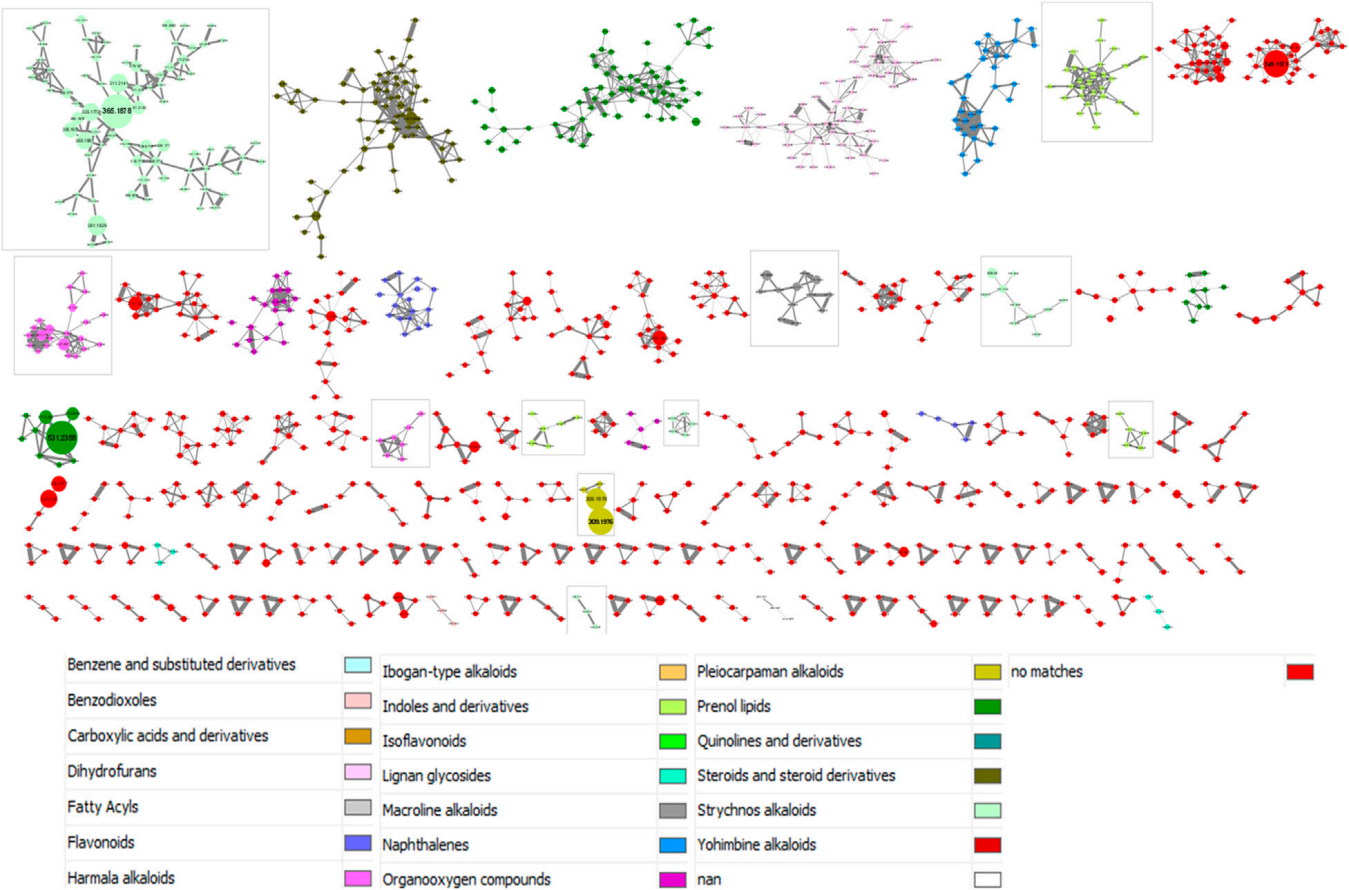
MolNetEnhancer-based annotation of the molecular network from 28 *Strychnos* species (Figure 2). The color legend is described below the molecular network. The framed clusters contain the phytochemical class of alkaloids. The size of nodes is related to the sum of precursor intensities

The results provided by the MolNetEnhancer workflow are shown in Figure 3. In this study, we interpreted the molecular network considering the class level. As in Figure 2, clusters with less than three ions have not been included in the figure for image size reasons. Moreover, in order to add a global quantitative aspect in the molecular network, the size of the nodes is related to the sum of precursor intensities. The larger the node, the greater the intensity of the precursor ion in question. A total of 11 clusters were categorized into the phytochemical class of alkaloids. These clusters were framed in Figure 3.

Nevertheless, some classifications of metabolites in clusters, proposed by the workflow, may be imprecise, or even faulty. This may be due to a misinterpretation of the workflow or to the presence of artifacts in the data. For example, we observed that strychnofoline, at *m/z* 483.2684, which is an oxindole monoterpenoid alkaloid, was assigned to the harmala alkaloids, presented in the Supplementary Figure S4. It is likely that this misclassification is related to the absence of the precise chemical class of strychnofoline in ClassyFire, a web-based application for automated structural classification of chemical entities (Djoumbou Feunang et al., 2016). For this reason, the application suggested the closest chemical class to strychnofoline, namely harmala alkaloids. Despite these inaccuracies, the classifications obtained are close to the expected result, and are therefore good indications for identifying metabolites.

**FIGURE 4:**
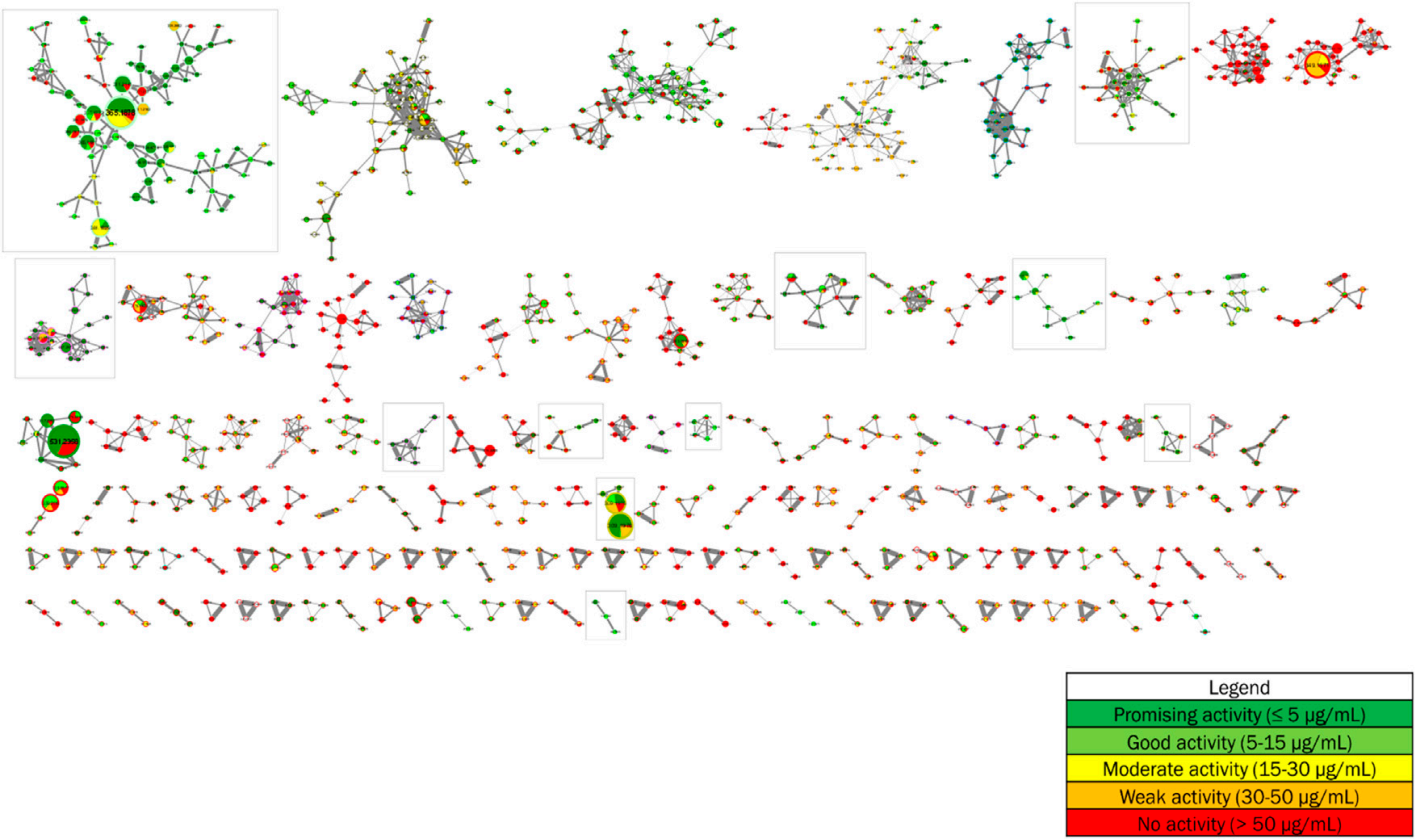
Implementation of antiplasmodial activities as metadata in the molecular network from 28 *Strychnos* species (Figure 2).

The antiplasmodial activities from the forthy-three methanolic crude extracts of *Strychnos*, described in point 3.2, were added as metadata to the global molecular network. These data, presented as pie charts within the nodes, allow to annotate the molecular network based on antiplasmodial activities and, therefore, to point to clusters and nodes with promising and good antiplasmodial activities. The proportion of different slices in the pie charts corresponds to the total intensities of metabolite constituting the node of interest within the different groups of antiplasmodial activities composed by the forty-three methanolic crude extracts according to the following color tags: promising (dark green), good (light green), intermediate (yellow), poor (orange) and/or absent (red) antiplasmodial activity (Figure 4).

Each of the eleven framed clusters contain a variety of known alkaloids, namely akuammicine, N^4^-methylantirhine, brucine, α-colubrine, corynantheidal, 6,7-dihydroflavopeirerine, icajine, malindine, isomalindine, C-mavacurine, naucleidinal, olivacine, panarine, retuline, strychnine, strychnofoline, strychnogucine C, sungucine, tubotaiwine, usambarensine, 3′,4′-dihydrousambarensine, N^b^-methylusambarensine, vincosamide, and vomicine. Moreover, some of these alkaloids, present mainly in extracts with promising or good activity, are related to ions of different masses still unknown to date and also present mainly in extracts with the same levels of antiplasmodial activity. However, it is important to note that an alkaloid, not identified in the molecular network, is not necessarily unknown in the literature. Indeed, identifications depend on databases that are not yet complete today. Enriching these databases would allow better identifications and even more efficient targeting of metabolites that were never reported in the literature.

**FIGURE 5:**
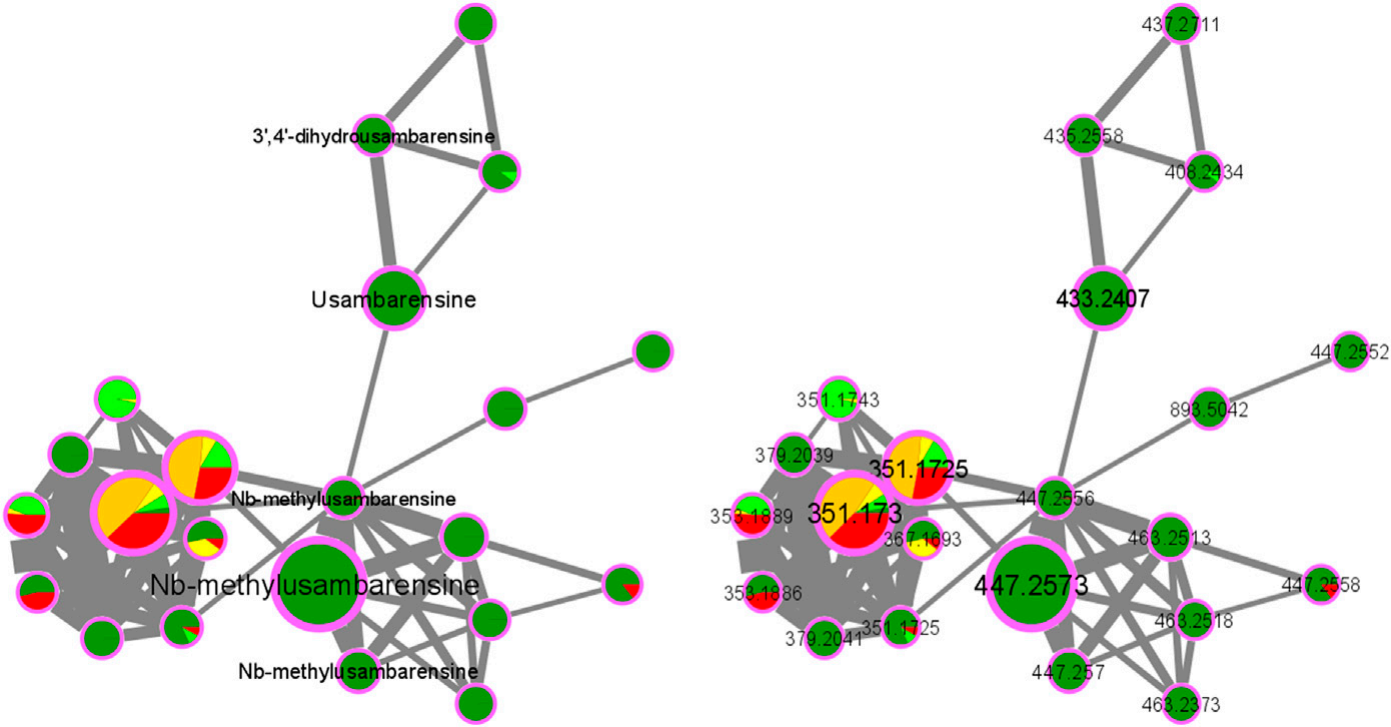
Alkaloids cluster n°3: Cluster of usambarensine and its derivatives.

**FIGURE 6:**
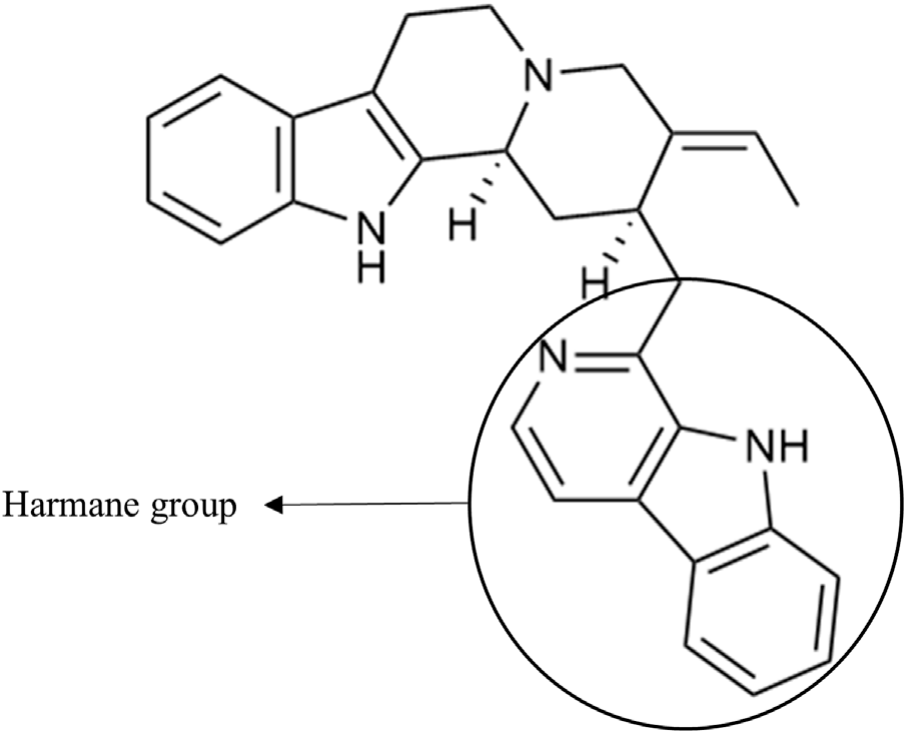
Structure of usambarensine.

As an example, the third framed cluster includes usambarensine, 3′,4′-dihydrousambarensine, and N^b^-methylusambarensine (Figure 5; Supplementary Table S3). The class assigned to this group is harmala alkaloids, which is confirmed because usambarensine and its derivatives include a harmane group in their structure (Figure 6). Well known in the *Strychnos* genus, especially for their promising antiplasmodial property against CQR and CQS strains of *P. falciparum*, usambarensine (CQS: 1.516 µM ± 0.031; CQR: 0.594 ± 0.052) and its derivatives are related to many non-identified ions, such as those at *m/z* 408.2434, 437.2711, 447.2552, 463.2373, 893.5042. A possible identification for the mass at *m/z* 437.2711 would be 1′,2′,3′,4′-tetrahydrousambarensine (Dictionary of Natural Products, 2022). About the other masses, no identification could be proposed. For more information, the Supplementary Table S4 includes all the different *m/z* values mentioned above, the generated molecular formulas used the mass spectrometry application software MassHunter (Version B.07.00), all suggested identifications provided by the Dictionary of Natural Products (Version 31.1) (Dictionary of Natural Products, 2022), the resulted interpretations, and the applied tolerances. Moreover, the nodes of all these ions are relatively small, which means that, based on the whole metabolites’ contents of the 43 methanolic crude extracts, the abundance of these ions is rather low. This observation would explain why the old techniques did not allow to visualize and identify these molecules of interest in previous research, especially on the leaves and roots of *S. usambarensis*.

**FIGURE 7:**
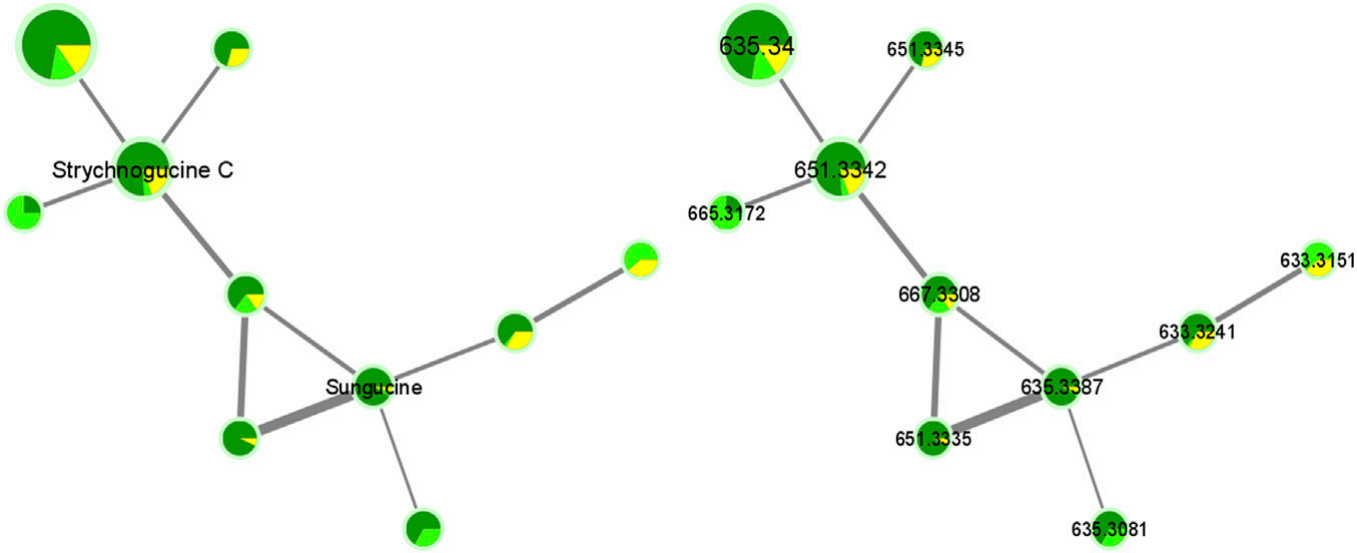
Alkaloids cluster n°5: Cluster of sungucine, and strychnogucine C.

As another example, we can cite the fifth framed cluster that belongs to the group of *Strychnos* alkaloids and that proposes strychnogucine C and sungucine as annotations (Figure 7; Supplementary Table S3). These two alkaloids are also well known in the *Strychnos* genus, especially sungucine, isolated for the first time from the roots of *S. icaja* and showed a promising antiplasmodial activity (CQS: 2.292 µM ± 0.049; CQR: 1.659 ± 0.089). Associated with these two alkaloids, different non-identified ions with masses at *m/z* 633.3241, 633.3151, 665.3172 and, 667.3308 are observed. The mass at *m/z* 667.3308 could correspond to strychnogucine B, which is a derivative of strychnogucine C. Moreover, several masses at *m/z* 635 and 651, corresponding to the masses of sungucine and strychnogucine C, respectively, are also present in the cluster. The differences in retention time and the low cosine values (from 0.67 to 0.70) lead rather to the hypothesis that they are different from each other, or even that they are isomers of sungucine and strychnogucine C. The ions at *m/z* 651.3335, and 651.3345, they could correspond to either strychnogucine A, which is also a derivative of strychnogucine C, or 18-hydroxysungucine, or 16,17-didehydro-17,23-dihydro-18-hydroxysungucine. All other masses, present in the fifth framed cluster, remain unknown (Supplementary Table S4).

Further investigation is needed to identify all the unknown alkaloids within the cluster. In addition, the sizes of the nodes show us that the abundances of the unknown ions are relatively low, except for the one with mass at *m/z* 635.3400 where the size is significantly larger. Indeed, its intensity is 10 times more important (Intensity at E8 compared to the other unknown ions that are at intensity E7).

For the other framed clusters, they are presented in the Supplementary Figures S1–S8. Among these clusters, one contains strychnofoline, which also shows interesting antiplasmodial activities, and is linked to three unidentified alkaloids (*m/z* 422.2317, 424.2404, and 440.2273) potentially active against malaria (Supplementary Tables S3, S4). A total of about 90 unknown alkaloids potentially active against malaria were detected using molecular networking technique. The advantage of this working method is therefore to target directly the unknown alkaloids, and, thus, to avoid wasting time to isolate already known alkaloids using the classical bioassay-guided fractionation method.

About the clusters in red, i.e., those where the MolNetEnhancer workflow did not provide a classification, it is important not to ignore them. Indeed, there may be a significant number of unknown ions that show interesting antiplasmodial activities. A closer look at these groups showed the presence of many masses above *m/z* 400. These masses could notably correspond to dimeric alkaloids that, in previous studies, demonstrated very good activities against *P. falciparum*.

Therefore, the development of mass spectrometry and chemoinformatic techniques, such as molecular networking, allows to have an overview of the metabolites’ content of the extracts studied, to quickly identify known ones, and to detect new potentially active ones, even with low quantities. The use of other dereplication tools such as ISDB (*In-Silico* spectral DataBase) (Allard, et al., 2016), MS2LDA (Wandy et al., 2018), MixONat (Bruguière et al., 2020), and MADByTE (Egan et al., 2021), and the realization of various bio- and mass-guided fractionations would allow to characterize and identify the new active metabolites observed in this study.

## Conclusion

Plants of *Strychnos* genus have fascinated researchers for more than two centuries because of their multiple traditional uses and their richness in metabolites, and more particularly in alkaloids, promising in the therapeutic field. Indeed, previous studies have highlighted various pharmacological activities of some species of the genus, especially against *Plasmodium* parasites, responsible for malaria, which continues to cause many deaths throughout the world, especially in Africa. In the face of growing parasite resistance, the current therapeutic arsenal is no longer sufficient to stop infections. Following the implementation of innovative chemoinformatic techniques such as molecular networking, an exploration of the alkaloid content of forty-three methanolic crude extracts from twenty-eight species of *Strychnos* was performed. In addition, these extracts were tested against the chloroquine-sensitive *P. falciparum* strain (3D7) in three independent test series. A total of 28% (12 extracts out of 43) showed promising (≤ 5 µg/ml) and good (between 5 and 15 µg/ml) antiplasmodial activities. The active extracts were from leaves and root barks of *S. usambarensis*, root barks of *S. variabilis*, leaves of *S. phaeotricha*, root barks of *S. angolensis*, trunk barks of *S. longicaudata*, leaves of *S. malchairi*, roots and collar barks of *S. icaja*, root barks of *S. nux-vomica*, and root barks of *S. malacoclados*. Some of these activities were never reported in the literature, namely these of trunk barks of *S. longicaudata* as well as leaves of *S. phaeotricha* and *S. malchairi*. These results, implemented as metadata in the molecular network of the forty-three methanolic crude extracts, allowed to highlight the presence of many alkaloids still unknown and potentially active against malaria. This is notably the case of alkaloids whose structures are close to those of usambarensine (at *m/z* 408.2434, 447.2552, 463.2373, 893.5042, …), sungucine and strychnogucine C, (at *m/z* 633.3241, 633.3151, 665.3172, 667.3308, …), strychnofoline (at *m/z* 422.2317, 424.2404, and 440.2273), and their corresponding derivatives, well known for their promising antiplasmodial properties.

In the future, it would be interesting to investigate further these still unknown alkaloids and to isolate them using bioassay- and mass-guided fractionations. The use of dereplication tools is also a way to obtain leads for the characterization and identification of these metabolites, even if they are present in small quantities.

Thus, this study demonstrates that the *Strychnos* genus still constitutes a significant therapeutic source and that the new approaches of dereplication offer new identification leads.

## Data Availability

The raw data supporting the conclusion of this article will be made available by the authors, without undue reservation.
