# Supplementary figures and images for: Unveiling antiplasmodial alkaloids from a cumulative collection of Strychnos extracts by multi-informative molecular networks
Source: Front Mol Biosci. 2022 Sep 26;9:967012. doi: 10.3389/fmolb.2022.967012 (PMC9548993; doi:10.3389/fmolb.2022.967012)

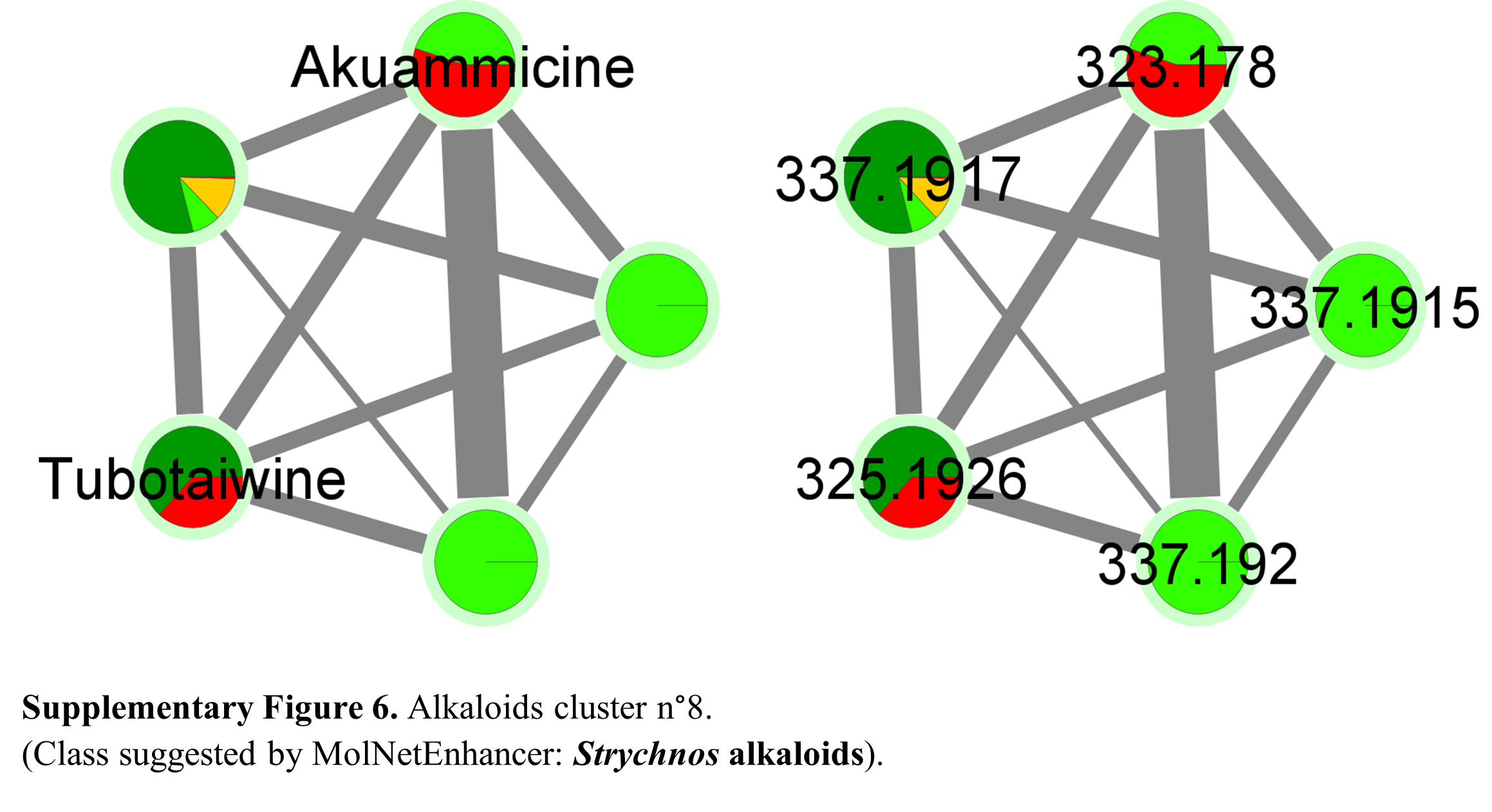

Supplement: Supplementary file 1 [file Image6.TIF]

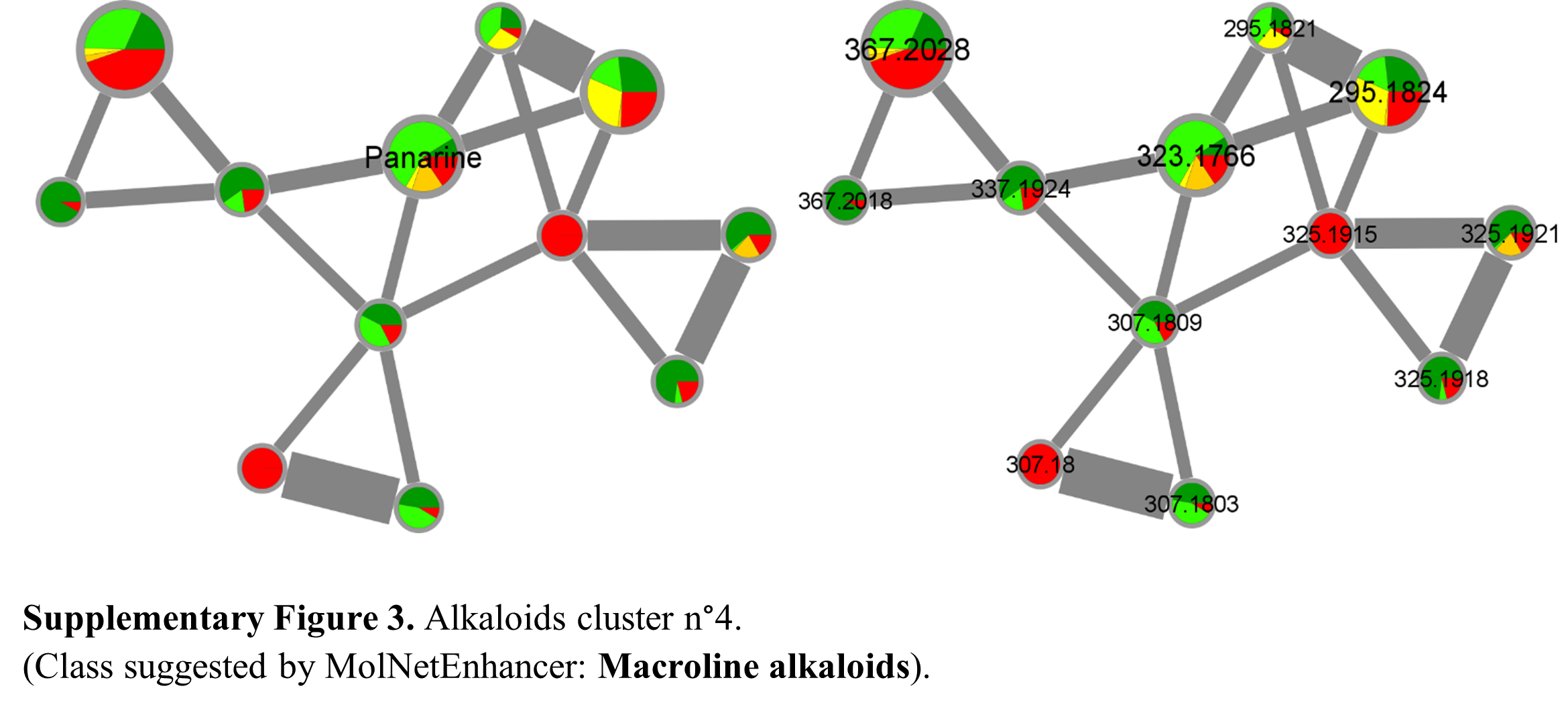

Supplement: Supplementary file 2 [file Image3.TIF]

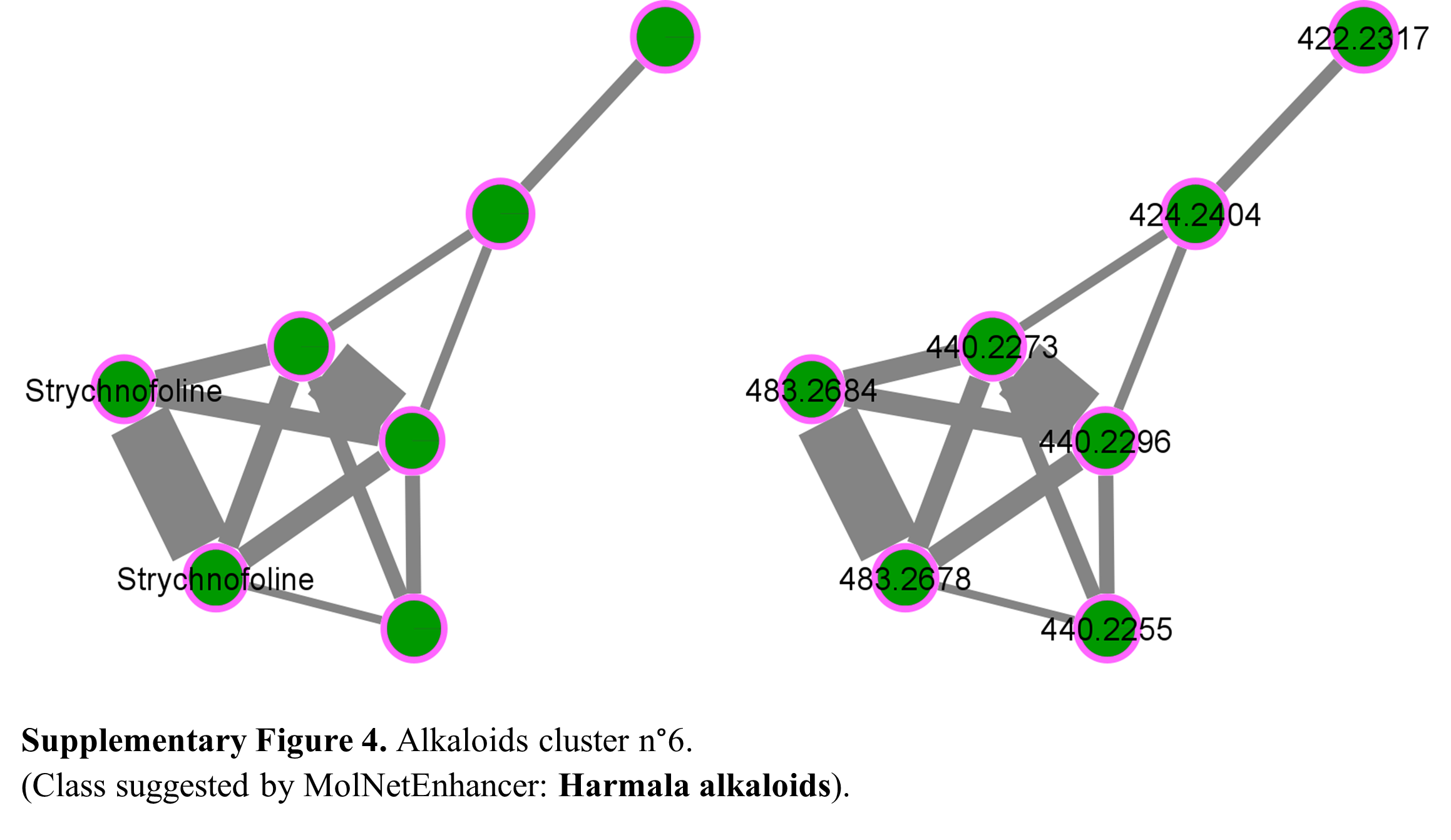

Supplement: Supplementary file 3 [file Image4.tif]

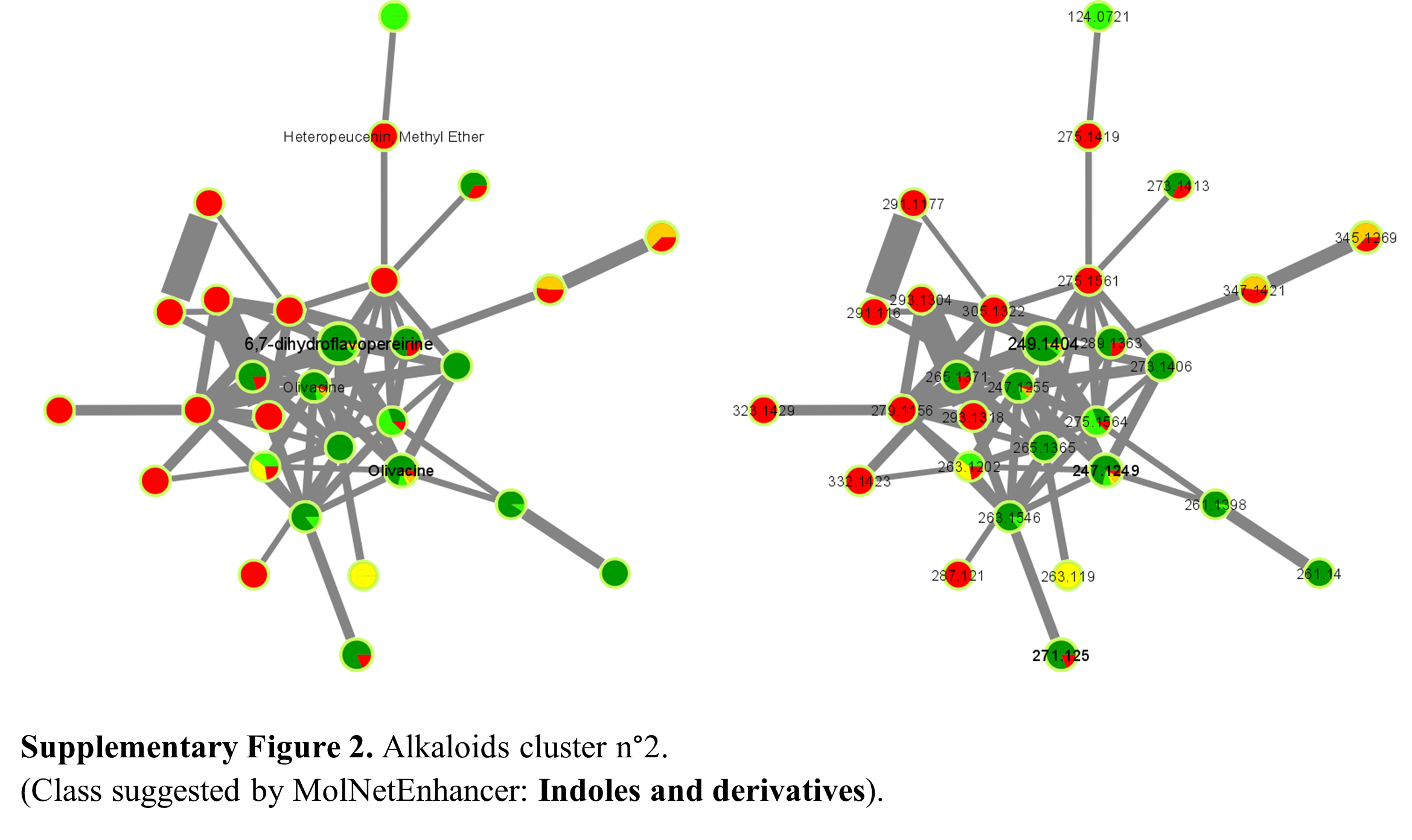

Supplement: Supplementary file 4 [file Image2.TIF]

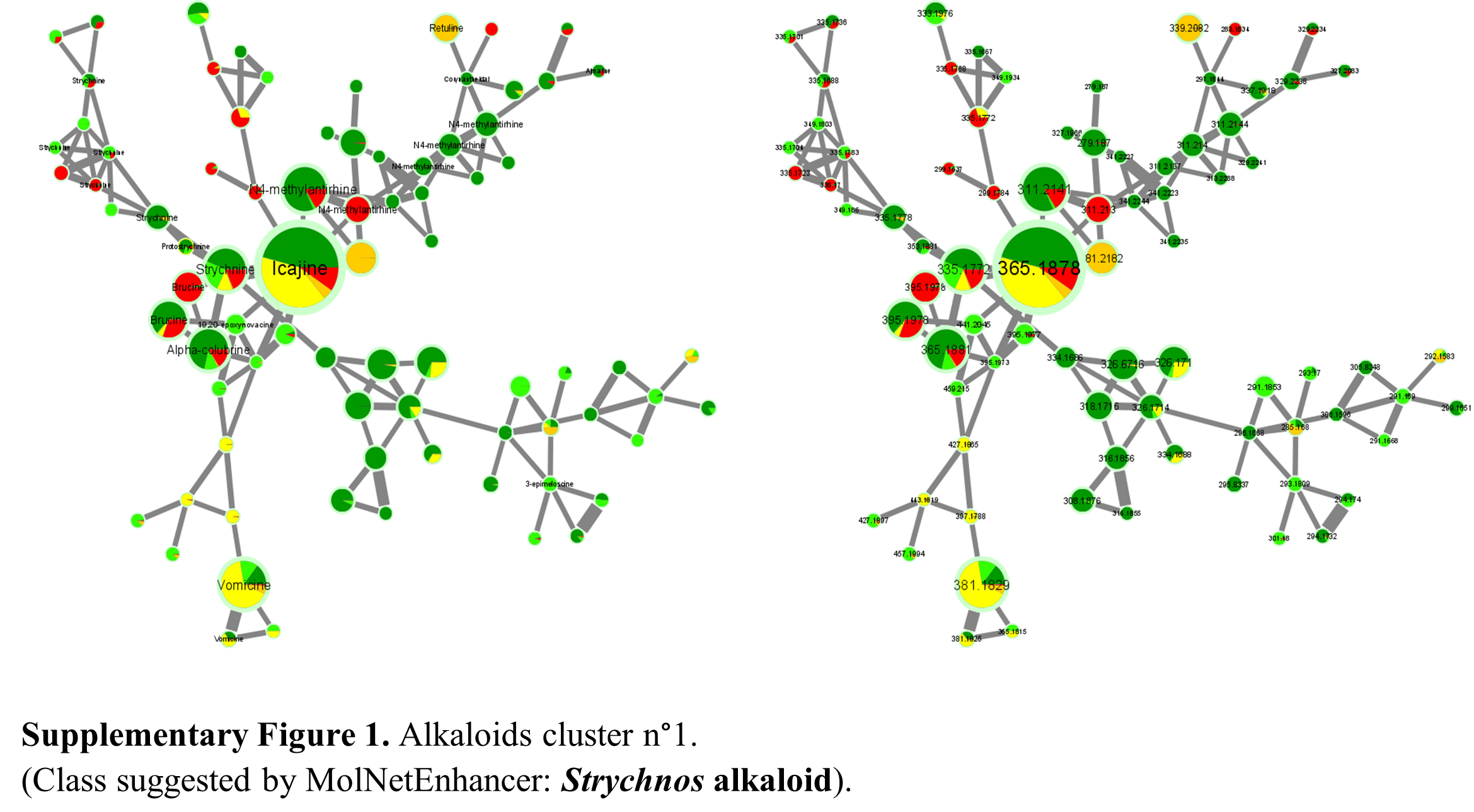

Supplement: Supplementary file 5 [file Image1.TIF]

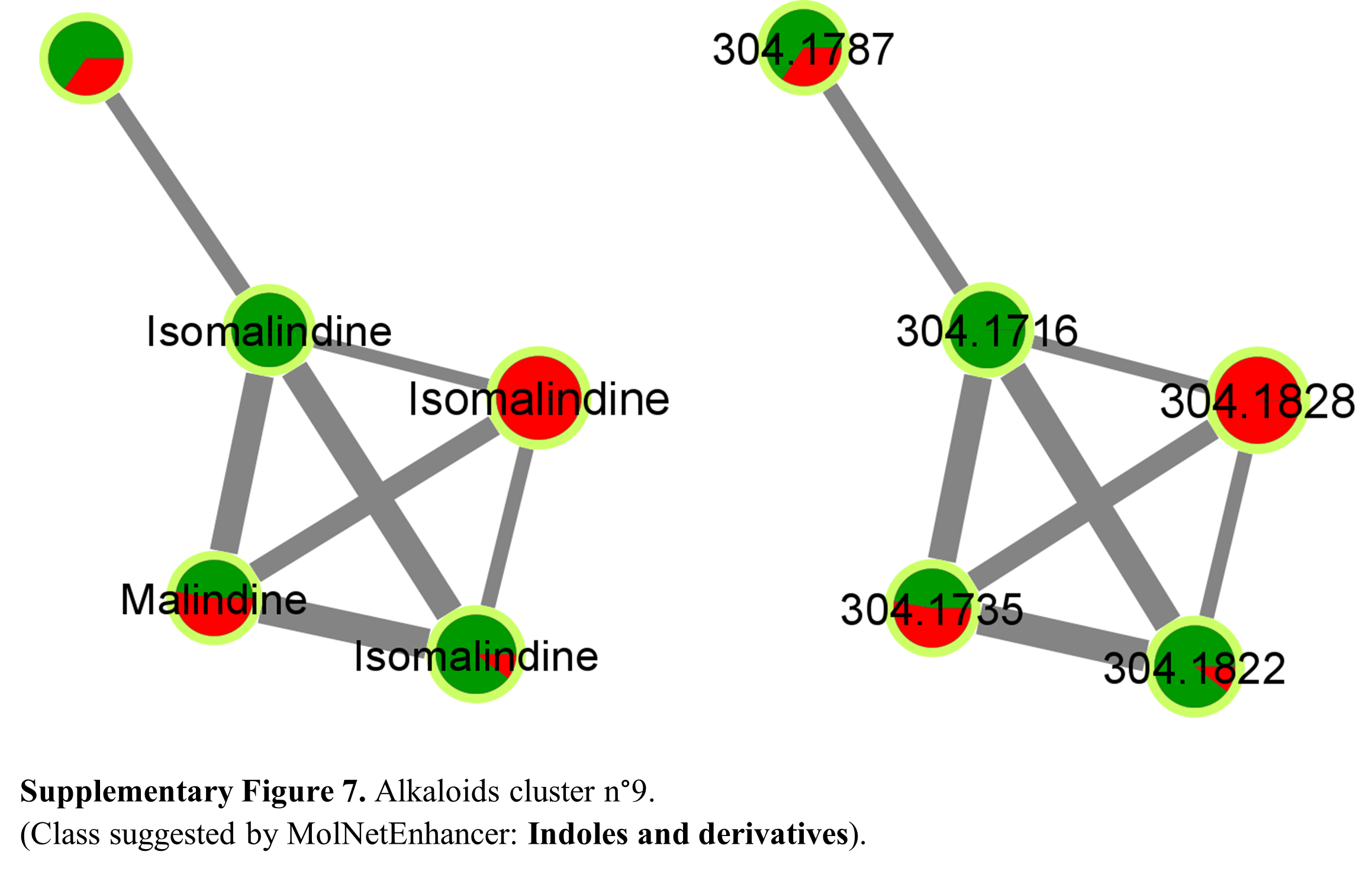

Supplement: Supplementary file 6 [file Image7.TIF]

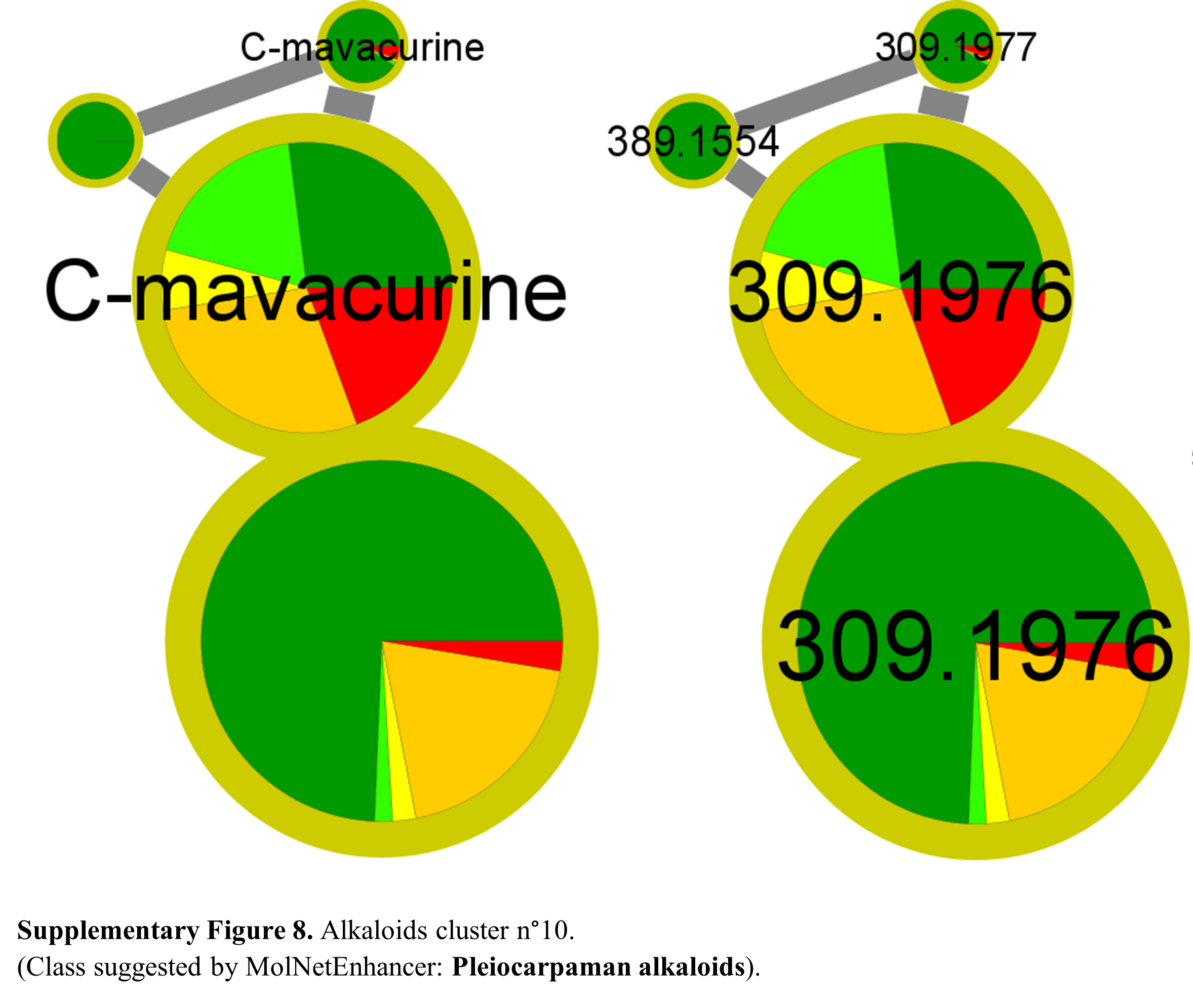

Supplement: Supplementary file 7 [file Image8.TIF]

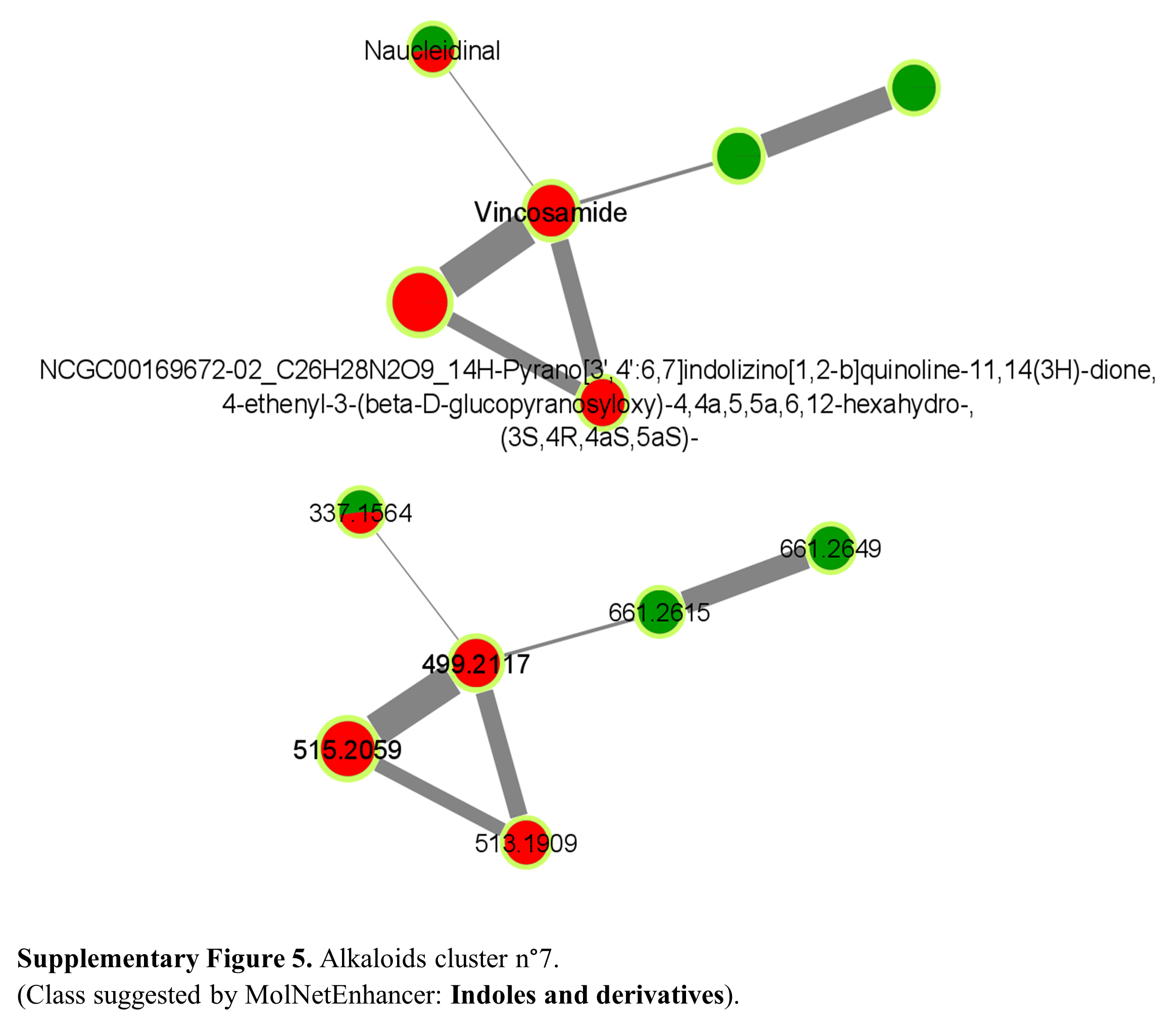

Supplement: Supplementary file 8 [file Image5.TIF]
