# Supplementary material for: Unveiling antiplasmodial alkaloids from a cumulative collection of Strychnos extracts by multi-informative molecular networks
Source: Front Mol Biosci. 2022 Sep 26;9:967012. doi: 10.3389/fmolb.2022.967012 (PMC9548993; doi:10.3389/fmolb.2022.967012)
Supplement: Supplementary file 9 [file DataSheet1.docx]

Supplementary Material

**Table of contents**

*Tables*

**Supplementary Table 1.** Origins of the *Strychnos* samples.

**Supplementary Table 2.** Weights and yields during the extractions of plants powder of *Strychnos* species.

**Supplementary Table 3.** Detailed MS data for usambarensine, 3',4'-dihydrousambarensine, N^b^-methylusambarensine, strychnogucine C, and strychnofoline.

**Supplementary Table 4.** Molecular formulas, and suggested identifications for the unannotated ions present in the framed clusters of usambarensine, sungucine as well as strychnogucine C, and strychnofoline.

## Supplementary Tables

**Supplementary Table 1.** Origins of the *Strychnos* samples.

| **Sample numbers** | **Names** | **Parts** | **Dates** | **Origins** | **Reference specimens** |
| --- | --- | --- | --- | --- | --- |
| 1 | *Strychnos usambarensis* Gilg ex Engl. | Leaves | November 2007 | Rwanda  (Akagera National Park, locality of Ndego) | Ulg20070608  (Herbarium of the Pharmaceutical Institute in Liège) |
| 2 |  |  | August 2008 | Rwanda  (Akagera National Park, locality of Ndego) | Ulg20080816  (Herbarium of the Pharmaceutical Institute in Liège) |
| 3 |  | Fruits | November 2007 | Rwanda  (Akagera National Park, locality of Ndego) | Ulg20070608  (Herbarium of the Pharmaceutical Institute in Liège) |
| 4 |  | Root barks | Oktober 1988 | Congo  (Locality of Luki) | Delaude HB3377  (Herbarium of Botanical Garden of Belgium at Meise) |
| 5 |  | Stem and twig barks | August 1948 | Congo  (Forest gallery in Kasenga) | Duvigneaud H1397  (Herbarium of Botanical Garden of Belgium at Meise and herbarium of the Pharmaceutical Institute in Liège) |
| 6 | *Strychnos variabilis* De Wild. | Root barks | 1970 | Congo  (Kinshasa, along the roads) | Evrard 6592  (Herbarium of Botanical Garden of Belgium at Meise) |
| 7 |  | Trunk barks | 1971 | Congo  (Kinshasa, Funa district) | Evrard 6592  (Herbarium of Botanical Garden of Belgium at Meise) |
| 8 | *Strychnos gossweileri* Exell | Root barks | 1994 | Congo  (near Matadi) | Delaude HB5690  (Herbarium of Botanical Garden of Belgium at Meise) |
| 9 | *Strychnos mellodora* S.Moore | Roots | August 1989 | Zimbabwe  (Chirinda Forest) | Delaude 7831  (Herbarium of Botanical Garden of Belgium at Meise) |
| 10 |  | Trunk barks | July 1997 | Congo | Delaude 7831  (Herbarium of Botanical Garden of Belgium at Meise) |
| 11 |  | Leaves | August 1989 | Zimbabwe  (Chirinda Forest) | Delaude 7831  (Herbarium of Botanical Garden of Belgium at Meise) |
| 12 | *Strychnos phaeotricha* Gilg | Leaves | Mai 1948 | Congo  (City of Kikwit) | Duvigneaud H914  (Herbarium of Botanical Garden of Belgium at Meise and herbarium of the Pharmaceutical Institute in Liège) |
| 13 | *Strychnos brasiliensis* (Spreng.) Mart. | Trunk barks | September 1997 | Brazil  (State of Parana, locality of Curitiba) | Cervi 6268  (Herbarium of the department of Botany from the Federal University of Parana |
| 14 | *Strychnos innocua* Delile | Leaves | August 2014 | Tanzania  (Kaole village of Bagamoyo district) | The reference specimen was deposited in the Herbarium unit of the department of Botany in the University of Dar es Salaam |
| 15 |  | Stem barks | July 1948 | Congo  (Locality of Keyberg) | Duvigneaud H1267  (Herbarium of Botanical Garden of Belgium at Meise and herbarium of the Pharmaceutical Institute in Liège) |
| 16 | *Strychnos henningsii* Gilg | Leaves | July 1948 | Congo  (Province of Katanga, between Tenge and Kolwezi) | Duvigneaud H1147  (Herbarium of Botanical Garden of Belgium at Meise and herbarium of the Pharmaceutical Institute in Liège) |
| 17 | *Strychnos angolensis* Gilg | Root barks | 1948 | Congo | Duvigneaud 1952  (Herbarium of Botanical Garden of Belgium at Meise) |
| 18 |  | Leaves | 1948 | Congo | Duvigneaud 1952  (Herbarium of Botanical Garden of Belgium at Meise) |
| 19 | *Strychnos scheffleri* Gilg | Leaves | June 1946 | Congo  (Forest in the north of Chikapa) | Duvigneaud H1088A  (Herbarium of Botanical Garden of Belgium at Meise and herbarium of the Pharmaceutical Institute in Liège) |
| 20 | *Strychnos tricalysioides* Hutch. & M.B.Moss | Trunk barks | November 1972 | Cameroon  (Near bridge in Loum-Kumba road, right bank of Mungo Creek) | Leeuwenberg 10604  WAG.1067021  (Herbarium Vadense at Wageningen*)  *The WAG herbarium moved to Leiden and is now part of the Naturalis Herbarium. |
| 21 | *Strychnos spinosa* Lam. | Leaves | August 2014 | Tanzania  (Kaole village of Bagamoyo district) | The reference specimen was deposited in the Herbarium unit of the department of Botany in the University of Dar es Salaam |
| 22 | *Strychnos longicaudata* Gilg | Roots and stems | April 1948 | Congo  (Locality of Kasongo-Lunda) | Duvigneaud H786b  (Herbarium of Botanical Garden of Belgium at Meise and herbarium of the Pharmaceutical Institute in Liège) |
| 23 |  | Trunk barks | August 2009 | Cameroon | Breteler F.J. 2135  Identified by comparison with the specimen of collection Herbarium n°9940 SRFCam at the National Herbarium of Cameroon  Kemeuze 7B  (Herbarium of Botanical Garden of Belgium at Meise) |
| 24 | *Strychnos malchairi* De Wild. | Leaves | June 1948 | Congo  (City of Panzi) | Duvigneaud H988  (Herbarium of the Belgian National Botanical Garden in Meise and herbarium of the Pharmaceutical Institute in Liège) |
| 25 |  | Trunk barks | June 1948 | Congo  (City of Panzi) | Duvigneaud H988  (Herbarium of the Belgian National Botanical Garden in Meise and herbarium of the Pharmaceutical Institute in Liège) |
| 26 | *Strychnos mattogrossensis* S.Moore | Leaves | June 1987 | Brazil  (near Manaus, Municipio do Careiro, Igarapé Grande do Lago do Rei) | COELHO, D. Herb. no. 142800  (Herbarium of INPA at Manaus, herbarium in the Department  of Plant Taxonomy, Agricultural University, Wageningen (Netherlands) and  herbarium of the Pharmaceutical Institute in Liège) |
| 27 | *Strychnos icaja* Baill. | Trunk barks | August 2009 | Cameroon  (6 km of Bertoua, on the road between Bertoua and Garoua Boulaï) | Breteler F.J. 1419  Identified by comparison with the specimen of collection Herbarium n°9934 SRFCam at the National Herbarium of Cameroon  Kemeuze 11B  (Herbarium of the Belgian National Botanical Garden in Meise) |
| 28 |  | Roots | June 2014 | Congo  (Province of Tshuapa, territory of Befale, locality of Lileko, in the equatorial forest) | Recolted by Phaacuen José Bayolo  Identified by chemical fingerprinting |
| 29 |  | Collar barks | May 1948 | Congo  (17 km from Kikwit to Leverville) | Duvigneaud H900  (Herbarium of Botanical Garden of Belgium at Meise and herbarium of the Pharmaceutical Institute in Liège) |
| 30 | *Strychnos nux-vomica* L. | Trunk barks | February 1999 | India  (State of Tamil Nadu, city of Chennai) | NV01  (Herbarium of the Pharmaceutical Institute in Liège) |
| 31 |  | Root barks | July 2007 | Cambodia  (Kampong Spoe Province) | SNVET.CaKS.07  Herbarium of the Faculty of Pharmacy in Marseille  Identified by Pr. Sun Kaing Cheng (botanist in the Faculty of Pharmacy of Phnom Penh, Cambodia) |
| 32 |  | Seeds | Oktober 2012 | Commercial sample  (Southeast Asia) | Commercial sample obtained from Denolin (Braine l’Alleud, Belgium) and Longeval (Deux Acres, Belgium)  Identified according to the description in the Swiss and French Pharmacopoeia |
| 33 | *Strychnos ignatii* P.J. Bergius | Fruits | November 1958 | Commercial samples (Southeast Asia) | Commercial sample obtained from Denolin (Braine l’Alleud, Belgium) and Longeval (Deux Acres, Belgium)  Identified according to the microscopic description in Perrot and Gathercoal (Gathercoal and Wirth, 1947 ; Perrot 1943) |
| 34 | *Strychnos potatorum* L.f. | Trunk barks | July 1948 | Congo  (Keyberg) | Duvigneaud H1202  (Herbarium of the Belgian National Botanical Garden in Meise and herbarium of the Pharmaceutical Institute in Liège) |
| 35 | *Strychnos malacoclados* C.H. Wright | Root barks | August 2009 | Cameroon  (6 km of Bertoua, on the road between Bertoua and Garoua Boulaï) | Kemeuze 14B  (Herbarium of the Belgian National Botanical Garden in Meise)  Identified at the National Herbarium of Cameroon |
| 36 | *Strychnos camptoneura* Gilg & Busse | Trunk barks | August 2009 | Cameroon | Breteler FJ 2572  Identified by comparison with the specimen of collection Herbarium n°9923 SRFCam at the National Herbarium of Cameroon |
| 37 | *Strychnos congolana* Gilg | Trunk barks | August 2009 | Cameroon | Letouzey 10555  Identified by comparison with the specimen of collection Herbarium n°23418 SRFCam at the National Herbarium of Cameroon |
| 38 | *Strychnos boonei* De Wild. | Trunk barks | August 2009 | Cameroon | Letouzey 1181  Identified by comparison with the specimen of collection Herbarium n°3427 SRFCam at the National Herbarium of Cameroon |
| 39 | *Strychnos* *staudtii* Gilg | Trunk barks | August 2009 | Cameroon | Nos 6105  Identified by comparison with the specimen of collection Herbarium n°31205 SRFCam at the National Herbarium of Cameroon |
| 40 | *Strychnos elaeocarpa* Gilg ex Leeuwenberg | Trunk barks | August 2009 | Cameroon | Bos 5405  Identified by comparison with the specimen of collection Herbarium n°31198 SRFCam at the National Herbarium of Cameroon |
| 41 | *Strychnos densiflora* Baill. | Trunk barks | August 2009 | Cameroon | Thomas D.W. 4286  Identified by comparison with the specimen of collection Herbarium n°53634 HNC at the National Herbarium of Cameroon |
| 42 | *Strychnos* *tchibangensis* Pellegr. | Trunk barks | August 2009 | Cameroon | Biholong M. 432  Identified by comparison with the specimen of collection Herbarium n°42104 SRFCam at the National Herbarium of Cameroon |
| 43 | *Strychnos* *johnsonii* Hutch. & M.B.Moss | Leaves | June 1948  (Identified in 1976 by Leeuwenberg) | Congo  (Panzi) | Duvigneaud H998  (Herbarium Vadense at Wageningen* and herbarium of the Pharmaceutical Institute in Liège)  *The WAG herbarium moved to Leiden and is now part of the Naturalis Herbarium. |

Each sample was dried at 40°C, stored dry at moderate room temperature, and protected from light. Some samples were collected several years ago. The question of the stability of alkaloids was therefore raised. Numerous previous studies of alkaloids showed a high stability (see point 2.1 in “Materials and methods” section). Oxidation reactions cannot be excluded, but no examples were highlighted in the different studies.

**Supplementary Table 2.** Weights and yields during the extractions of plants powder of *Strychnos* species.

| **Names** | **Parts and reference specimens** | **Weights** | **Types of extract** | **Yields** |
| --- | --- | --- | --- | --- |
| *Strychnos usambarensis* | Leaves  (Ulg20070608) | 5.44 g | MeOH | 15.63 % |
|  |  | 2.00 g | Alkaloidic | 4.000 % |
|  | Leaves  (Ulg20080816) | 5.44 g | MeOH | 25.92 % |
|  | Fruits  (Ulg20070608) | 5.44 g | MeOH | 13.05 % |
|  | Root barks  (Delaude HB3377) | 5.44 g | MeOH | 6.800 % |
|  | Stem and twig barks  (Duvigneaud H1397) | 5.44 g | MeOH | 15.81 % |
| *Strychnos variabilis* | Root barks  (Evrard 6592) | 5.44 g | MeOH | 0.5500 % |
|  | Trunk barks  (Evrard 6592) | 5.44 g | MeOH | 3.310 % |
| *Strychnos gossweileri* | Root barks  (Delaude HB5690) | 5.44 g | MeOH | 13.97 % |
| *Strychnos mellodora* | Roots  (Delaude 7831) | 5.44 g | MeOH | 9.190 % |
|  | Trunk barks  (Delaude 7831) | 5.44 g | MeOH | 2.570 % |
|  | Leaves  (Delaude 7831) | 5.44 g | MeOH | 6.610 % |
| *Strychnos phaeotricha* | Leaves  (Duvigneaud H914) | 5.44 g | MeOH | 6.620 % |
| *Strychnos brasiliensis* | Trunk barks  (Cervi 6268) | 5.44 g | MeOH | 11.76 % |
| *Strychnos innocua* | Leaves  (Deposited in the Herbarium unit of the department of Botany in the University of Dar es Salaam) | 5.44 g | MeOH | 23.16 % |
|  | Stem barks  (Duvigneaud H1267) | 5.44 g | MeOH | 7.720 % |
| *Strychnos henningsii* | Leaves  (Duvigneaud H1147) | 5.44 g | MeOH | 13.97 % |
| *Strychnos angolensis* | Root barks  (Duvigneaud 1952) | 5.44 g | MeOH | 9.010 % |
|  | Leaves  (Duvigneaud 1952) | 5.44 g | MeOH | 5.330 % |
| *Strychnos scheffleri* | Leaves  (Duvigneaud H1088A) | 5.44 g | MeOH | 8.640 % |
| *Strychnos tricalysioides* | Trunk barks  (Leeuwenberg 10604) | 5.44 g | MeOH | 0.7400 % |
| *Strychnos spinosa* | Leaves  (Deposited in the Herbarium unit of the department of Botany in the University of Dar es Salaam)) | 5.44 g | MeOH | 25.00 % |
| *Strychnos longicaudata* | Roots and stems  (Duvigneaud H786b) | 5.44 g | MeOH | 3.860 % |
|  | Trunk barks  (Breteler F.J. 2135) | 5.44 g | MeOH | 5.510 % |
| *Strychnos malchairi* | Leaves  (Duvigneaud H988) | 5.44 g | MeOH | 6.620 % |
|  | Trunk barks  (Duvigneaud H988) | 5.44 g | MeOH | 6.070 % |
| *Strychnos mattogrossensis* | Leaves  (COELHO, D. Herb. no. 142800) | 5.44 g | MeOH | 0.9200 % |
| *Strychnos icaja* | Trunk barks  (Breteler F.J. 1419) | 5.44 g | MeOH | 9.190 % |
|  | Roots  (Recolted by Phaacuen José Bayolo) | 5.44 g | MeOH | 9.930 % |
|  | Collar barks  (Duvigneaud H900) | 5.44 g | MeOH | 9.190 % |
| *Strychnos nux-vomica* | Trunk barks  (NV01) | 5.44 g | MeOH | 8.270 % |
|  | Root barks  (SNVET.CaKS.07) | 5.44 g | MeOH | 4.230 % |
|  | Seeds  (Commercial sample) | 5.44 g | MeOH | 6.800 % |
| *Strychnos ignatii* | Fruits  (Commercial sample) | 5.44 g | MeOH | 7.170 % |
| *Strychnos potatorum* | Trunk barks  (Duvigneaud H1202) | 5.44 g | MeOH | 8.460 % |
| *Strychnos malacoclados* | Root barks  (Kemeuze 14B) | 5.44 g | MeOH | 1.650 % |
| *Strychnos camptoneura* | Trunk barks  (Breteler FJ 2572) | 5.44 g | MeOH | 0.7400 % |
| *Strychnos congolana* | Trunk barks  (Letouzey 10555) | 5.44 g | MeOH | 3.490 % |
| *Strychnos boonei* | Trunk barks  (Letouzey 1181) | 5.44 g | MeOH | 0.550 % |
| *Strychnos staudtii* | Trunk barks  (Nos 6105) | 5.44 g | MeOH | 4.230 % |
| *Strychnos elaeocarpa* | Trunk barks  (Bos 5405) | 5.44 g | MeOH | 2.870 % |
| *Strychnos densiflora* | Trunk barks  (Thomas D.W. 4286) | 5.44 g | MeOH | 2.760 % |
| *Strychnos tchibangensis* | Trunk barks  (Biholong M. 432) | 5.44 g | MeOH | 2.570 % |
| *Strychnos johnsonii* | Leaves  (Duvigneaud H998) | 5.44 g | MeOH | 13.42 % |

**Supplementary Table 3.** Detailed MS data for usambarensine, 3',4'-dihydrousambarensine, N^b^-methylusambarensine, strychnogucine C, and strychnofoline.

| **Metabolites** | **Raw formulas** | **Parent masses (*m/z*)** | **Calculated masses (*m/z*)** | **Retention times (minutes)** | **MQScores** | **MZErrorPPM (ppm)** |
| --- | --- | --- | --- | --- | --- | --- |
| Usambarensine | C_29_H_29_N_4_ [M+H^+^] | 433.2407 | 433.5674 | 14.0327 | 0.863232 | 4.57864 |
| 3’,4’-Dihydrousambarensine | C_29_H_31_N_4_ [M+H^+^] | 435.2558 | 435.5832 | 13.2740 | 0.845967 | 3.43560 |
| N^b^-Methylusambarensine | C_30_H_31_N_4_^+^ [M^+^] | 447.2556 | 447.5939 | 14.4427 | 0.842087 | 2.93403 |
| Sungucine | C_42_H_43_N_4_O_2_ [M+H^+^] | 635.3387 | 635.8164 | 13.7428 | 0.783390 | 0.960672 |
| Strychnogucine C | C_42_H_43_N_4_O_3_ [M+H^+^] | 651.3342 | 651.8158 | 14.5877 | 0.747934 | 1.87416 |
| Strychnofoline | C_30_H_35_N_4_O_2_ [M+H^+^] | 483.2684 | 483.6245 | 11.2923 | 0.833754 | 14.7133 |

**Supplementary Table 4.** Molecular formulas, and suggested identifications for the unannotated ions present in the framed clusters of usambarensine, sungucine as well as strychnogucine C, and strychnofoline. Molecular formulas are generated from MassHunter (Version B.07.00), and suggested identifications are provided by the Dictionary of Natural Products (Version 31.1).

| ***m/z*** | **Molecular formulas** | **Hits** | **Comments** |
| --- | --- | --- | --- |
| 408.2434 | C_16_H_33_N_5_O_7_ | Neomycin A; 5-Deoxy, 1-*N*-(4-amino-2*S-*hydroxybutanoyl) | Unknown identification in the *Strychnos* genus but this is unlikely. |
| 422.2317 | C_21_H_31_N_3_O_6_ | Trichopeptide B | Unknown identification in the *Strychnos* genus but this is unlikely. |
|  | C_26_H_31_NO_4_ | Ancistrobrevine A; Atropisomer, 6-*O*-de-Me  Ancistrobrevine A; 6-*O*-De-Me  Ancistrobrevine B; Atropisomer, *N*-Me  Ancistrobrevine B; 1-Epimer, 4'-*O*-de-Me, 6-Me ether, *N*-Me  Ancistrobrevine B; 6-Me ether  Ancistrocladine; Atropisomer, 1-epimer, Me ether  Ancistrocladine; Atropisomer, Me ether  Ancistrocladine; 1-Epimer, *N*-Me  Ancistrocladine; 1- and/or 3-Epimer, Me ether  Ancistrocladine; Me ether  Ancistrorobertsonine D; Atropisomer, 8-*O*-de-Me, *O*^6^,*N*-di-Me  Ancistrorobertsonine D; 8-*O*-De-Me, *O*^6^,*N*-di-Me  Ancistrorobertsonine D; 1-Epimer, 6-Me ether  Ancistrorobertsonine D; *N*-Me  Ancistrorobertsonine D; 6-Me ether  Ancistrotectorine  Ancistrotectorine A  Ancistrotectorine C  Antibiotic TMC 69  Korupensamine A; Atropisomer, 5',8-di-Me ether, *N*-Me  Korupensamine A; 5',6-Di-Me ether, *N*-Me  Lythrumine  Militarinone D | Unknown identifications in the *Strychnos* genus. |
| 424.2404 | C_16_H_33_N_5_O_8_ | Neomycin A; 1-N-(4-Amino-2S hydroxybutanoyl) | Unknown identification in the *Strychnos* genus but this is unlikely. |
| 437.2711 | C_29_H_32_N_4_ | 2,13-Diethyl-3,7,8,12,17-pentamethyl-21H,23H-porphine  2,13-Diethyl-3,7,12,17-tetramethyl-21H,23H-porphine; 18-Methyl  2,13-Diethyl-3,7,12,17-tetramethyl-21*H*,23*H*-porphine; 20-Methyl  Ochrolifuanines; (3*R*,17ξ,20ξ)(1)-form, 18,19-Didehydro  Ochrolifuanines; (3*R*,17ξ,20ξ)(2)-form, 18,19-Didehydro  Ochrolifuanines; (3S,17R,20R)-form, 18,19-Didehydro  2,3,7,8,12,13,17,18-Octamethylporphin; 2-Ethyl analogue  Usambarensine; 1'R,2',3',4'-Tetrahydro  Usambarensine; 1'S,2',3',4'-Tetrahydro | It is likely that the mass at *m/z* 437.2711 corresponds to 1',2',3'4'-tetrahydrousambarensine. |
| 440.2273 | C_18_H_29_N_7_O_6_ | Glutamylhistidylglycylvaline | Unknown identification in the *Strychnos* genus but this is unlikely. |
|  | C_22_H_33_NO_8_ | Echiumine; 3'-Epimer, 1'''-hydroxy, 3'-Ac  Echiumine; 3'-Epimer, 1'''-hydroxy, 5'-Ac  Heliosupine; 3'-Ac  Hydrocotyline  Leptanthine; 3'- or 5'-Angeloyl, 7-Ac  Parsonsianidine; 14-Deoxy  Parsonsine | Unknown identification in the *Strychnos* genus. |
| 447.2552 | No hit | | Unknown metabolite. |
| 463.2373 | C_31_H_30_N_2_O_2_ | Cimicifoetone A; 1,1-Dimethyl-2-propenyl analogue | Unknown identification in the *Strychnos* genus. |
| 633.3241 | No hit | | Unknown metabolite. |
| 633.3151 | No hit | | Unknown metabolite. |
| 651.3335 | C_25_H_46_N_8_O_12_ | Antibiotic B 3454 | Unknown identification in the *Strychnos* genus but this is unlikely. |
|  | C_42_H_42_N_4_O_3_ | Strychnogucine A  Strychnogucine A; Δ17',23'-Isomer  Sungucine; 18-Hydroxy  Sungucine; 18-Hydroxy, 16,17-didehydro, 17,23-dihydro | It is likely that the mass at *m/z* 651.3335 corresponds to either strychnogucine A or 18-hydroxysungucine or 16,17-didehydro-17,23-dihydro-18-hydroxysungucine. |
| 651.3345 | C_25_H_46_N_8_O_12_ | Antibiotic B 3454 | Unknown identification in the *Strychnos* genus but this is unlikely. |
|  | C_42_H_42_N_4_O_3_ | Strychnogucine A  Strychnogucine A; Δ17',23'-Isomer  Sungucine; 18-Hydroxy  Sungucine; 18-Hydroxy, 16,17-didehydro, 17,23-dihydro | It is likely that the mass at *m/z* 651.3345 corresponds to either strychnogucine A or 18-hydroxysungucine or 16,17-didehydro-17,23-dihydro-18-hydroxysungucine. |
| 665.3172 | C_40_H_44_N_2_O_7_ | 7,8-Epoxy-3,12(18)-dolabelladiene-2,10,15-triol; (2β,7α,8β,10β)-form, 2,10-Bis-O-(3-pyridinecarbonyl), 15-phenylacetyl | Unknown identification in the *Strychnos* genus but this is unlikely. |
| 667.3308 | C_42_H_42_N_4_O_4_ | Strychnogucine B | It is likely that the mass at *m/z* 667.3308 corresponds to strychnogucine B. |
| 893.5042 | No hit | | Unknown metabolite. |

The margins of error of masses were 2.0 mDa (5.6 ppm) for MS, and 5.0 mDa (7.5 ppm) for MS/MS.
